# Stakeholder Perspectives on Digital Health Technologies in Poststroke Self-Management and Rehabilitation: Systematic Review Based on the NASSS Framework

**DOI:** 10.2196/95197

**Published:** 2026-10-09

**Authors:** Jing Zhao, Jie Zhao, Xiaohan Liu, Yongxin Cao, Xuan Wang, Yanhong Dong, Hong Chang

**Affiliations:** 1 Department of Neurology, Xuanwu Hospital, Capital Medical University, No. 45 Changchun Street, Beijing, 100053, China, 86 13701034090; 2 Alice Lee Centre for Nursing Studies, Yong Loo Lin School of Medicine, National University of Singapore, Singapore

**Keywords:** stroke, digital health technologies, stroke care, systematic review, self-management, adoption

## Abstract

**Background:**

Digital health technologies (DHTs) offer opportunities to support poststroke self-management and rehabilitation, but their implementation may be hindered by mismatches between technological design, stakeholder needs, and care contexts. Synthesizing stakeholder perspectives can clarify implementation priorities and gaps in the evidence.

**Objective:**

This study aimed to synthesize stakeholders’ perceptions, experiences, and recommendations regarding DHT implementation in poststroke self-management and rehabilitation and to map the findings to the NASSS (Nonadoption, Abandonment, Scale-Up, Spread, and Sustainability) framework.

**Methods:**

Six databases (PubMed, MEDLINE, Embase, Scopus, CINAHL, and Web of Science) were searched from inception through October 2025. Eligible studies were English-language, peer-reviewed primary studies using qualitative, quantitative, or mixed methods designs to report stakeholder perspectives on DHTs in poststroke care. Two reviewers independently screened studies, extracted data, and assessed methodological quality using the Mixed Methods Appraisal Tool (MMAT). A convergent integrated synthesis was conducted: quantitative findings were transformed into textual descriptors, combined with qualitative findings, coded inductively, and subsequently mapped deductively to NASSS domains and subdomains. Disagreements were resolved through consensus or by third-reviewer adjudication.

**Results:**

Of 15,262 records identified, 42 studies published between 2019 and 2025 were included: 24 qualitative, 8 quantitative, and 10 mixed methods studies. Patients, health care professionals, caregivers, and members of the public were represented in 32, 19, 8, and 3 studies, respectively, with some studies including multiple groups. Evidence concentrated on the condition, technology, value proposition, and adopter-system domains. Stakeholders valued personalized functions, intuitive interfaces, timely feedback, and digital support that complemented human care. Barriers included stroke-related motor, cognitive, communication, and sensory impairments; unequal digital access and literacy; caregiver demands; staff workload and training needs; and poor integration with clinical routines. Data privacy and unclear payment arrangements also raised concerns. Evidence on organizational readiness and wider-system determinants was limited, and no eligible study directly examined interaction and adaptation over time. Methodological concerns included nonresponse bias and insufficient interpretation of integrated findings in mixed methods studies.

**Conclusions:**

Stakeholder perspectives indicate that DHT implementation in poststroke care should combine accessible, personalized design with support for patients, caregivers, and health care professionals, alongside integration into clinical workflows and clear data governance and financing arrangements. The NASSS mapping identified priorities beyond usability, but current evidence does not establish sustained adoption, scale-up, or spread. Longitudinal studies in routine care are needed to evaluate adaptation and sustainability.

## Introduction

### Background

Stroke remains a major public health challenge worldwide and a leading contributor to mortality and disability [1]. According to the 2021 Global Burden of Disease (GBD) study, there were 93.8 million prevalent cases of stroke, 11.9 million new stroke events, and 7.3 million deaths from stroke [2]. Without strengthened prevention, treatment, and long-term care strategies, the global stroke burden is projected to continue increasing substantially by 2050 [3]. Nearly half of patients who are poststroke experience long-term disabilities that compromise independence and quality of life, resulting in ongoing needs for rehabilitation, self-management support, and long-term care [4].

The rapid development and adoption of digital health technologies (DHTs) have created unprecedented opportunities for supporting chronic disease management and home-based rehabilitation [5]. DHTs refer to technology-enabled tools that support health information delivery, monitoring, and care management, such as patient portals, mobile apps, wearable devices, and telemedicine platforms [6]. By making care more accessible and flexible and potentially reducing costs, DHTs could extend stroke support beyond traditional clinical settings and facilitate clinical decision-making, personalized care, and service delivery for populations with diverse needs, including those in low-resource settings [7].

DHTs are increasingly used to support poststroke monitoring, self-management, rehabilitation, and remote consultations [8]. Growing evidence suggests that digital health interventions can improve several clinically relevant outcomes among patients who are poststroke. For example, digital health–enabled stroke management has been associated with improved blood pressure control and reduced stroke recurrence [9]. In addition, meta-analytic evidence indicates potential benefits for poststroke cognitive functioning [10]. Home-based mobile health interventions have also shown potential to support risk-factor control and continuity of poststroke care [11]. By offering alternative and personalized interventions, DHTs can help overcome barriers to care and mitigate workforce shortages. DHTs may offer opportunities for scale-up, although successful dissemination and maintenance of intervention fidelity depend on the implementation context and sustained organizational support [12].

DHTs can deliver their potential benefits only when they are successfully implemented in clinical practice [7,13]. Real-world implementation requires not only evidence of clinical effectiveness but also a clear understanding of how DHTs are perceived, adopted, and integrated by relevant stakeholders [7]. Stakeholder perspectives from patients, caregivers, health care professionals (HCPs), organizations, funders, and policymakers are therefore essential for identifying the practical needs, contextual barriers, and system-level conditions that shape the implementation of DHTs in stroke care [14]. The NASSS (Nonadoption, Abandonment, Scale-Up, Spread, and Sustainability) framework was developed to support the evaluation and implementation of technology-supported health care programs [15]. Its seven domains cover the condition, technology, value proposition, adopters, organization, wider system, and interaction and adaptation over time, providing a structured lens for analyzing the implementation complexity of DHTs in stroke care [15,16].

### Objectives

An increasing number of primary studies have examined the use of DHTs to support self-management and rehabilitation in patients who are poststroke [17-20]. However, few systematic reviews have synthesized this evidence through a structured implementation framework such as the NASSS framework. Applying the NASSS framework can help identify implementation determinants across diverse clinical and care settings and support more comparable, theory-informed implementation research [13]. The aims of this mixed methods systematic review were (1) to explore the perceptions, experiences, and recommendations of key stakeholders regarding the implementation of DHT in poststroke self-management and rehabilitation and (2) to map these findings onto the NASSS framework. This review provides implementation-oriented evidence to guide the future integration and scaling of DHT interventions in stroke care.

## Methods

### Search Strategy

We followed the PRISMA (Preferred Reporting Items for Systematic Reviews and Meta-Analyses) guidelines [21]. The review protocol was registered in PROSPERO (International Prospective Register of Systematic Reviews; CRD420251112158). We searched 6 databases (PubMed, MEDLINE, Embase, Scopus, CINAHL, and Web of Science) from inception through October 2025. Reviews were excluded, but their reference lists and those of eligible studies were checked for additional records. Controlled vocabulary and free-text terms were tailored to each database. The full search strategies for all databases are available in Multimedia Appendix 1.

### Eligibility Criteria

DHTs were defined as technology-based interventions that use data collection, automated analysis, feedback, or remote communication to support rehabilitation or disease management [8]. Studies were eligible if they (1) investigated a DHT, including therapy or education app, digital rehabilitation services, automated reminders, self-administered online services, or objective monitoring tools; (2) involved patients with stroke or stakeholders relevant to their care, including patients, family caregivers, family members, and HCPs; (3) reported qualitative or quantitative evidence on stakeholder perspectives, experiences, recommendations, or implementation challenges; and (4) were published in English. The intended end users had to include patients with stroke. We excluded technologies used only to deliver therapist-led exercises without data storage, feedback, or interactive functionality and communication-only services (eg, email, chat, or videoconferencing) without an autonomous or scalable digital component.

### Study Selection

After deduplication in EndNote X9 (version 9.3.3; Clarivate Analytics), 2 reviewers independently screened the titles and abstracts of retrieved articles from the search strategy that potentially adhered to the study eligibility criteria. Full texts were then reviewed by the 2 reviewers for inclusion in the review. Disagreements were resolved through discussion and, when consensus could not be reached, through adjudication by a third reviewer.

### Quality Appraisal

Two reviewers independently appraised the included studies using the 2018 Mixed Methods Appraisal Tool (MMAT) [22]. No study was excluded on the basis of the MMAT appraisal, and no overall numerical quality score or statistical weighting was applied. Criterion-level methodological concerns were considered when interpreting the breadth and strength of the NASSS-mapped findings.

### Data Extraction

A shared Microsoft Excel extraction form, aligned with Joanna Briggs Institute guidance, was piloted on a subset of studies and refined before full extraction [23]. Two reviewers independently extracted publication details, country, design, data collection and analysis methods, participant characteristics, setting, DHT types, implementation context, and stakeholder findings. Discrepancies and unclear reporting were discussed by 3 authors, and the agreed decisions were recorded in the final extraction file.

### Data Synthesis and Integration

We used a convergent integrated synthesis to analyze qualitative, quantitative, and mixed methods findings together [24]. Quantitative results were transformed into textual descriptors that retained the direction, magnitude, population, and context of the original result; qualitative findings and mixed methods results were extracted as author interpretations supported, where available, by participant quotations. The analysis proceeded in 3 stages. First, 2 reviewers independently applied open codes to the integrated findings without using the NASSS framework as an a priori coding structure. Second, related codes were compared and refined inductively into clinically meaningful categories through team discussion. Third, these categories were mapped deductively to the 7 NASSS domains and their subdomains using MAXQDA 2020 (VERBI Software) [25]. The NASSS framework was selected because it provides a common structure for comparing interacting implementation determinants across heterogeneous technologies, stakeholders, and care contexts [16,26,27]. As NASSS was originally developed primarily for evaluating individual implementation cases, we used it as an organizing framework. A category could be mapped to more than 1 domain when it represented distinct mechanisms. Findings that did not fit an existing subdomain were retained and used to develop 2 adapted technology subdomains. Disagreements were resolved through consensus or adjudication by a third reviewer.

## Results

### Literature Search Results

The searches identified 15,262 records. After 5492 duplicates were removed, 9770 records underwent title and abstract screening, and 269 articles were assessed in full text. Of these, 227 were excluded and 42 studies were included (Figure 1): 24 qualitative studies [17,18,20,28-48], 8 quantitative studies [19,49-55], and 10 mixed methods studies [56-65]. Devittori et al [55,66] published related studies in 2024 and 2025. Because the latter extended the former, only the 2024 study was included in the synthesis [55]. One included study used NASSS in its primary analysis [31]. Four stakeholder groups were represented: patients with stroke, caregivers, HCPs (including physicians, nurses, occupational therapists, and physiotherapists), and members of the public.

**Figure 1.**
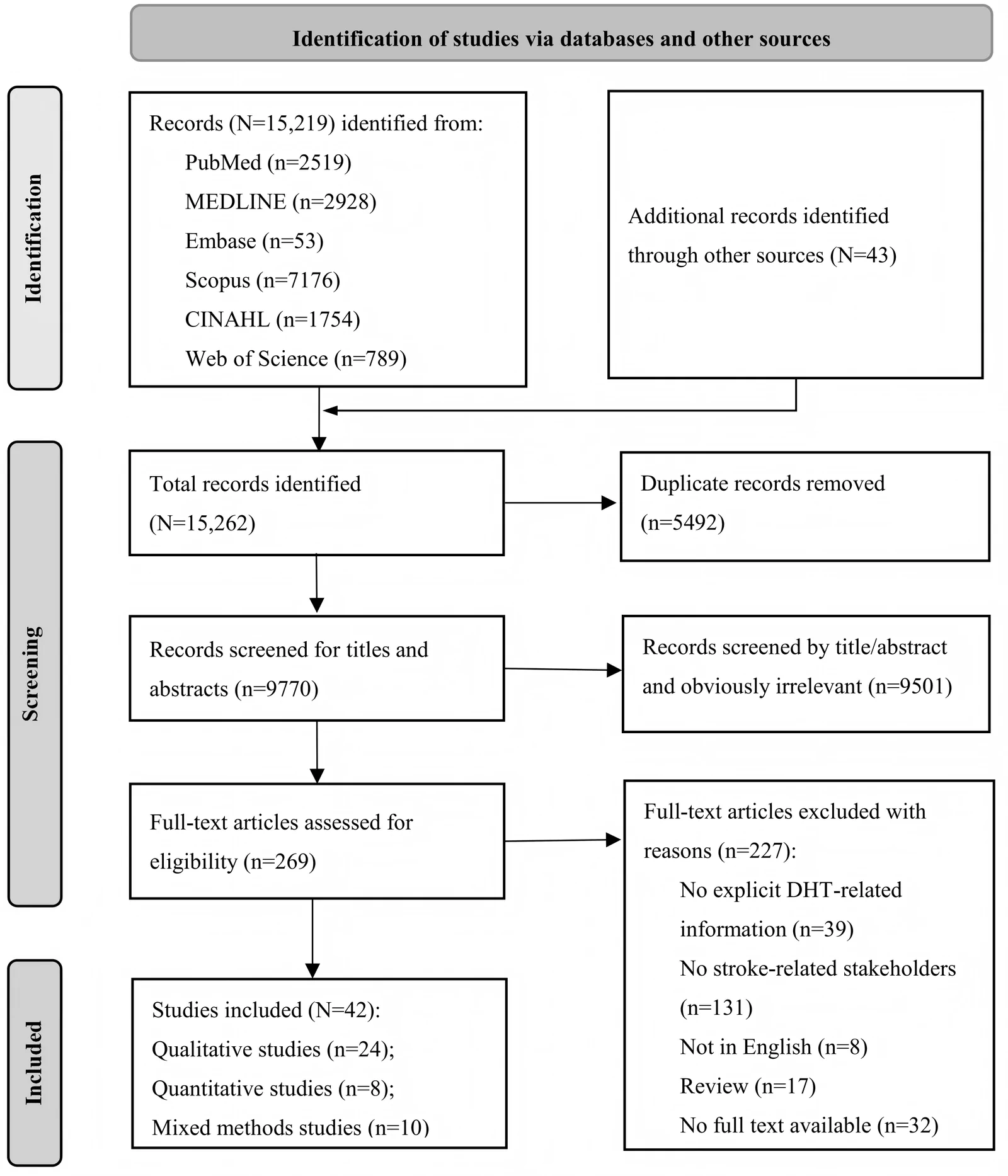
PRISMA (Preferred Reporting Items for Systematic Reviews and Meta-Analyses) flowchart of the systematic review. DHT: digital health technologies.

### Study Characteristics

Detailed characteristics of the 42 included studies are presented in Multimedia Appendix 2. The included studies were published between 2019 and 2025, with the number of publications increasing from 2022 onward. Studies were conducted in 21 countries: Australia (n=7), the United States (n=5), the United Kingdom (n=4), and China (n=3) were the most represented, while the remaining 23 studies were distributed across 17 countries. Settings included hospitals (n=14), rehabilitation centers (n=8), communities (n=4), homes (n=2), combined hospital-community settings (n=2), and multiple settings (eg, homes, hospitals, and communities; n=4). Two studies were conducted online, and 1 analyzed published user reviews of commercial stroke apps. The remaining 5 studies reported only geographical coverage without specifying the study setting. Six studies reported no funding, 18 received national research funding, 8 received institutional or association grants, 5 reported other funding, and 5 did not report funding.

### Participant Characteristics

Of the 42 studies, 32 included patients, 19 included HCPs, 8 included caregivers, and 3 included members of the public. Because individual studies could include more than one stakeholder group, these stakeholder-study counts exceed 42. Sixteen of the 32 patient-related studies included only patients with stroke. By study design, the 24 qualitative studies included 441 participants, the 8 quantitative studies included 830 participants, and the 10 mixed methods studies included 1550 participants. Overall, studies reported 1197 men and 1319 women participants. Gender was not reported for 305 participants. Sample sizes ranged from 4 to 48 for qualitative studies, 13 to 486 for quantitative studies, and 19 to 1194 for mixed methods studies. Technologies included mobile apps, wearable devices, telemedicine platforms, virtual reality systems, rehabilitation robots, and AI-enabled tools used for remote monitoring, personalized rehabilitation, and disease management.

### Methodological Quality of Included Studies

Quality appraisal results are detailed in Multimedia Appendix 3. Among the 24 qualitative studies, most showed coherence between their aims, data collection, analysis, and interpretation, although 2 did not clearly report data-collection procedures [29,44]. Among the 8 quantitative studies, measures and analyses were generally appropriate; 5 had a low risk of nonresponse bias [49-52,55], whereas 3 had a higher or unclear risk because response rates or differences between respondents and nonrespondents were insufficiently reported [19,53,54]. Only 4 of the 10 mixed methods studies adequately interpreted the outputs of qualitative-quantitative integration [56,58,60,63].

### Themes

#### Overview

Inductive content analysis generated clinically meaningful categories, which were subsequently mapped to the NASSS domains and subdomains across the 42 studies. Evidence was concentrated in the condition, technology, value proposition, and adopter-system domains. No relevant evidence was identified for organizational capacity to innovate, the nature of organizational adoption or funding decisions, the professional context, the sociocultural context at the wider system level, or interaction and adaptation over time. Two adapted subdomains were added within the material features to distinguish personalization and accessibility from usability and simplicity. Figure 2 summarizes facilitators, barriers, and evidence gaps across the 7 domains. Figure 3 shows the study-level distribution of stakeholder evidence.

**Figure 2.**
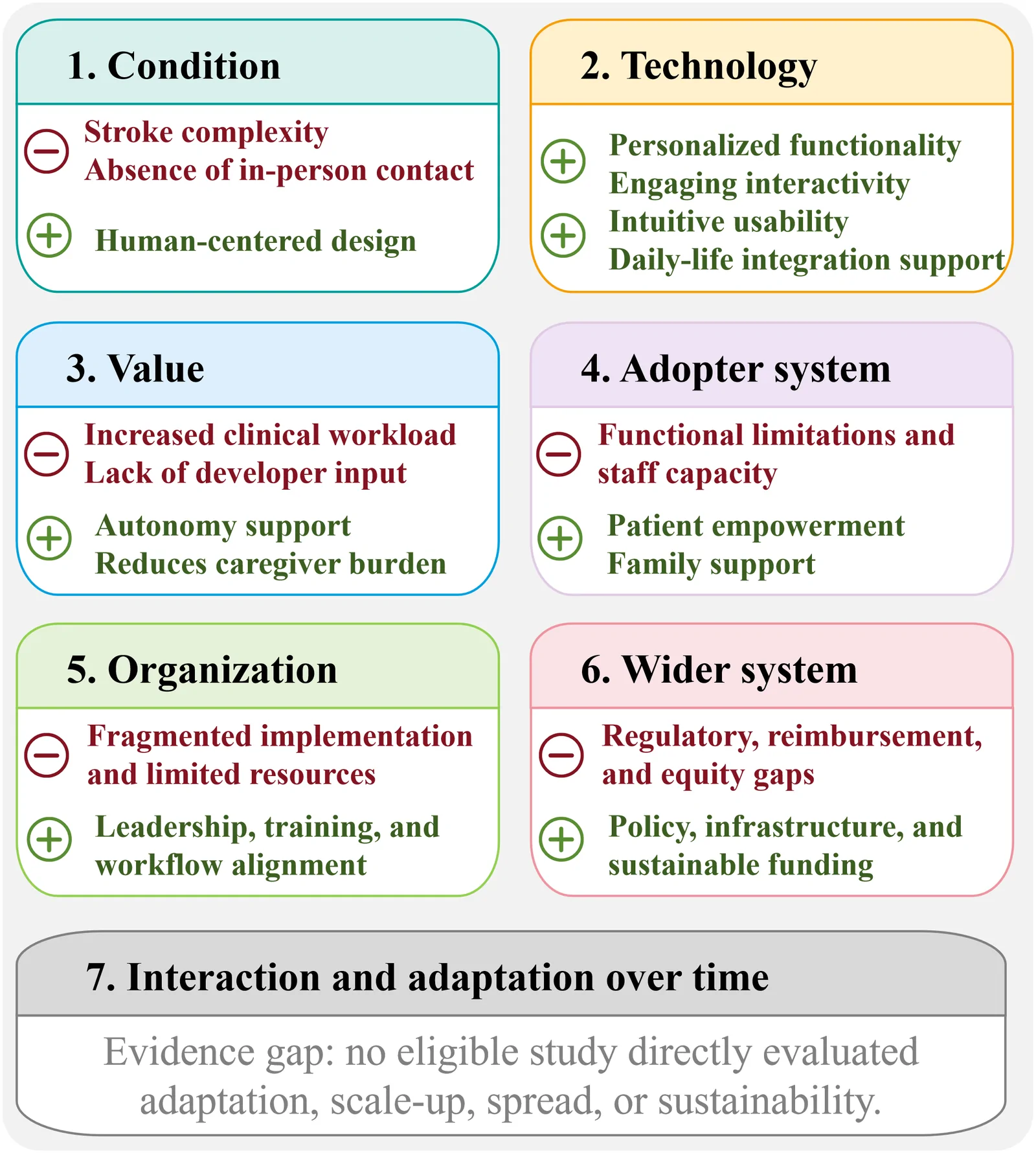
Synthesis of implementation determinants across the 7 NASSS (Nonadoption, Abandonment, Scale-Up, Spread, and Sustainability) domains. Green plus signs denote facilitators and red minus signs denote barriers reported by stakeholders. Domain 7 is shown in gray because no eligible study directly examined adaptation, scale-up, spread, or sustainability. The facilitators and barriers shown summarize key findings rather than providing an exhaustive list; detailed findings are presented in the Results section.

**Figure 3.**
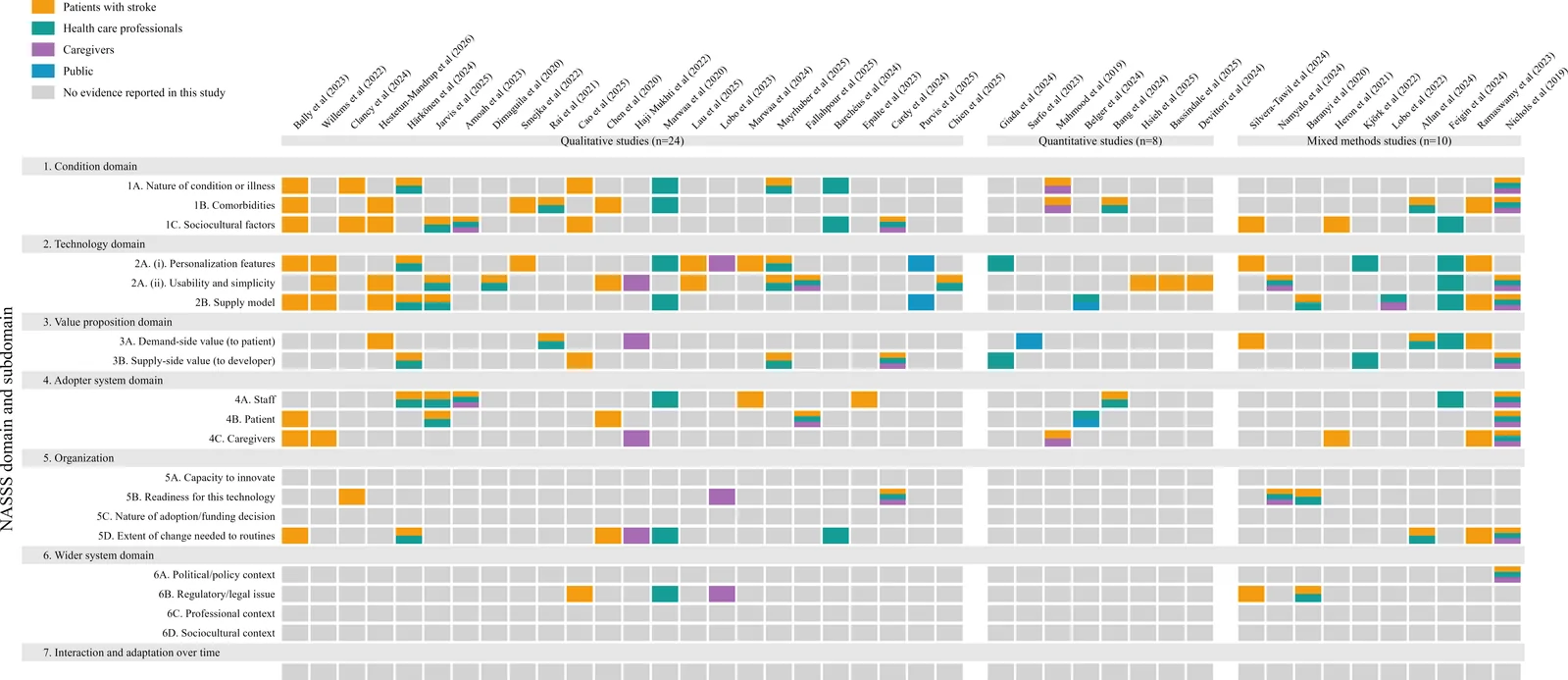
Matrix summarizing stakeholder perspectives reported across NASSS (Nonadoption, Abandonment, Scale-Up, Spread, and Sustainability) domains and subdomains. Columns represent 42 studies grouped by design, and rows represent NASSS domains and subdomains. Stacked colors indicate multiple stakeholder groups within the same study and subdomain [17-20,28-65].

#### Condition Domain

The first NASSS domain focuses on the illness-related context in which an intervention or technology is implemented. Across the included studies, stakeholders emphasized the context-dependent nature of DHTs, highlighting how the characteristics of stroke, together with the scope and setting of implementation, shaped technological requirements and constraints. Consideration of the stroke or clinical context was reported in 22 studies.

##### Nature of Condition or Illness

Across the included studies, perspectives on DHTs were predominantly patient-centered, reflecting the complex and heterogeneous nature of stroke [48]. Stroke-related impairments, including motor, cognitive, communication, emotional, visual, attentional, and fatigue-related difficulties, were frequently reported to constrain patients’ capacity to engage with digital technologies and self-management activities [30,35]. Stakeholders viewed DHTs as supportive tools to facilitate poststroke self-management and rehabilitation rather than as standalone solutions [18]. The long-term and evolving recovery trajectory of stroke was identified as a challenge for the implementation of DHTs. While digital support was increasingly regarded as necessary to sustain rehabilitation and ongoing care beyond the acute phase [38,44], multiple studies emphasized that patients remained central to their recovery journey, with technology serving a complementary role [20]. Maintaining person-centered care, preserving human connection, and tailoring the balance between digital and face-to-face support to individual needs were consistently identified as key facilitators of effective implementation in poststroke contexts [42,51].

##### Comorbidities

Stroke commonly co-occurs with other chronic conditions [36]. Common comorbidities include cognitive, memory, language, and attention deficits, as well as psychomotor, fine motor, and sensory impairments [18,38]. Motivation to use DHTs was influenced by the perceived severity and relevance of the target problem, such as sleep disturbance [33]. Management of hypertension, diabetes, HIV/AIDS, and coronary heart disease was also considered important [34]. As noted by a participant, “Stroke is often a consequence of other chronic diseases such as hypertension, diabetes, HIV/AIDS, and coronary heart disease. Many with these underlying risk factors remain at risk if not well managed” [57]. Barriers therefore extended beyond clinical impairment to limited digital literacy, inadequate home infrastructure, and low motivation [29,34]. Many studies excluded people with moderate-to-severe cognitive impairment or aphasia, which limits the representativeness and generalizability of the evidence [19,20,52].

##### Sociocultural Factors

Stroke recovery demands longitudinal, human-centered technologies rather than one-off interventions [40]. Patients, caregivers, and clinicians consistently regarded in-person contact as important for safety, respect, and therapeutic relationships [18,20,35]. One patient noted: “It has to be personal by talking to each other in person and not via video call” [18]. Digital rehabilitation could not replace human interaction and care [29]. Acceptance was facilitated when DHTs complemented face-to-face care, incorporated culturally appropriate features, and involved family members [29,31]. Moreover, some HCPs have expressed concerns that DHTs could be mistaken for a substitute for clinical input, raising fears of professional displacement [44]. Acceptance of technology is further influenced by age, cultural norms such as familial caregiving expectations, and disability-related stigma [56]. Older adults required interfaces adapted to physical, cognitive, and social needs [35]. In younger adults, the reported underuse concerned stroke-specific digital tools rather than general digital literacy; limited age-targeted design and the perception that existing tools were intended for older populations may reduce relevance and engagement [17].

### Technology Domain

The technology domain refers to the materials, data, knowledge, and supply-related characteristics of DHTs. Most included studies (30/42, 71.4%) primarily focused on proposed functions, technical features, and user interface design. Based on the findings, 2 key aspects related to material characteristics were identified. In contrast, issues concerning knowledge development and intellectual property were addressed far less frequently.

#### Personalization Features

Material features of DHTs were reported as facilitators. Personalization was a central material feature of DHTs. It addressed heterogeneity in patients’ impairments, goals, and recovery trajectories [18,28], particularly through adaptable interface design and prioritization of core functions [64]. Features such as adjustable text size, zoom functions, and simplified layouts were perceived as enhancing accessibility for users with sensory or motor impairments [40,42]. Establishing an individualized information library enables autonomous retrieval of relevant information, covering topics like stroke causes, medication, prognosis, and follow-up care [18,49]. In addition, providing opportunities to explore advanced information through external resources and reliable project-specific websites is considered beneficial [47]. Patient-entered health data enabled tailored alerts and reminders [30,33,40,56]. Continuous customized support can maintain motivation, self-management, and empowerment [41]. Personalization also enhances user-friendliness and promotes long-term adherence to ambulatory monitoring [47,60,64]. The interactive features such as chatbots, scoring, rewards, animations, and motivational toolkits were also considered important to successful DHT design [38,39].

#### Usability and Simplicity

Across studies, simplicity and intuitive navigation were central to adoption. Patients preferred systems that required little cognitive effort and remained stable, free from advertisements and disruptive updates [18,28]. As an HCP noted, “Keep it as simple as possible; these are all terms that people, especially with cognitive impairments, find difficult to understand” [28]. Stable interfaces with clear navigation, free from advertisements and disruptive updates, were regarded as essential for usability and cognitive accessibility [38]. “I think it needs to be easy to use. Some people I find are surprisingly lazy, and if it’s not easy to use the first time, they just won’t go back and try again,” a health expert noted [47]. Systems integrating medication reminders, activity-based goal setting, structured daily routines, and self-monitoring functions (eg, symptoms, sleep, and physical activity) within user-friendly diaries or checklists were perceived as highly relevant and supportive of daily use [30,31]. Incorporating stroke education and rehabilitation guidance boosted relevance and immersion [29]. These features increased the likelihood that a technology could be incorporated into everyday life rather than used only during a short study period [31].

#### Supply Model

Across studies, relevant and timely feedback was identified as a central facilitator of engagement with DHT [31,32]. DHTs commonly generated personalized behavioral reports [28], providing feedback on progress, adherence, and outcomes [19,54]. Patients particularly valued positive and corrective feedback to sustain motivation and to make slow recovery trajectories visible [42]. One patient noted, “I need more feedback. If I have doubts, I can use it as a reference” [37]. DHTs generated behavioral health reports based on individualized health data and on users’ performance [55]. Patients received feedback after submitting their daily performance scores [43]. As a patient explained, “Tracking your recovery so that you can actually see or know you’re recovering. It’s such a slow process. You want something that gives you feedback each day on how you’re going” [17]. Clinicians also relied on real-time or near-real-time feedback to monitor patient activity and inform follow-up, valuing automated updates that required minimal manual oversight. For example, staff using the VERA (Virtual Engagement Rehabilitation Assistant) system valued automatic updates: “as long as it gives me feedback, like how many times this patient exercised over the weekend” [31]. Feedback delivered through visual [39], auditory [55], interactive [54], and rapid real-time formats [19] was associated with improved user experience, with rapid feedback and embedded encouragement further supporting motivation and emotional resilience [19,36,54].

### Value Proposition Domain

The value proposition domain concerns whether the technology is worth developing or introducing for patients, clinicians, and suppliers. Across stakeholder groups, DHTs were broadly perceived as adding value beyond usual care, particularly in enhancing efficiency, accuracy, and accessibility [49].

#### Demand-Side Value (to the Patient)

Patients reported that DHTs could improve education, monitoring, and access to follow-up support. Quantitative research has indicated that more than 80% of users are satisfied with the technical performance and perceived health benefits of DHTs [50,56]. Patients valued reminders for home exercises, timely health information, access to therapists, and the ability to monitor progress [63,64]. Nevertheless, trust in clinicians and health care organizations generally exceeded trust in digital systems, and infrequent phone use, limited flexibility, and low perceived treatment relevance reduced demand [56]. Caregivers anticipated reduced workload [37], whereas HCPs were concerned that poorly designed monitoring and messaging systems could generate anxiety and additional clinical work [34].

#### Supply-Side Value (to the Developer)

Developer-related perspectives were reported in 7 studies, but they mainly came from HCPs involved in co-design rather than from commercial technology developers. Systematically eliciting the “voice of stroke patients,” prioritizing requirements, and providing reliable educational resources were considered essential [31,40]. From the demand-side perspective, patients similarly emphasized that developers ensure easy access to reliable educational resources [35]. Automation was valued for providing consistent monitoring and prompts, but it did not remove the need for clinical oversight, validation, or accountability [29]. The absence of direct industry perspectives limited conclusions about intellectual property, commercial incentives, interoperability, and scalability.

### Adopter System Domain

Clinicians, patients, and family caregivers were identified as key stakeholder groups whose roles are expected to evolve with the adoption of DHTs. The adopter domain focuses on these groups’ capacity, motivation, and willingness to adopt and sustain the use of DHTs.

#### Staff

Within the adopter system, HCPs linked patient-generated data with clinical decision-making. Medical staff emphasized the importance of timely, transparent access to patient data and digital resources as a prerequisite for engagement [31]. DHTs enable clinicians to monitor patients’ health status longitudinally, manage structured clinical records, and use decision-support tools to guide clinical care [30]. These capabilities supported phased goal setting, remote monitoring, and continuity of care during transitions to community-based rehabilitation teams [17]. However, barriers to adoption were also prominent. Many clinicians reported high workload, limited training and digital literacy, and insufficient organizational support [38]. Clinicians were also reluctant to recommend technologies that lacked public funding, institutional approval, or clear evidence of benefit [41].

#### Patient

Intuitive, adaptable, and intrinsically motivating DHTs facilitated patients’ transition from passive care recipients to active self-managers and increased awareness of their health status and recovery progress [32]. Patients valued the autonomy to determine when and how to engage with DHTs, which influenced both adoption and sustained use [43]. DHTs also supported home-based recovery by promoting independence in daily activities and rehabilitation, thereby fostering a sense of control that was often less evident in clinic-centered care [38]. This emerging digital awareness reflected patients’ increasing recognition of their poststroke capabilities and energy limitations in everyday life. However, physical, cognitive, and emotional consequences of stroke, together with fatigue and low confidence in using DHTs, constrained sustained engagement for some patients [20,45].

#### Caregivers

Family caregivers were important implementation partners who provided technical assistance, emotional encouragement, and structure for daily routines. Family and peer support could reduce isolation and reinforce engagement [28]. However, caregivers also faced limited digital literacy, distrust of technology, competing demands, and family tension [18]. Sustainable implementation therefore requires explicit assessment of caregiver capacity, training and support for those who choose to participate, and safeguards against transferring clinical or technical responsibilities to families without adequate resources [28,37].

### Organization

This domain addresses organizational capacity, readiness, adoption decisions, and the changes required to integrate DHTs into routine stroke care. Evidence was reported in 10 qualitative and 4 mixed methods studies but was limited to readiness for this technology and the extent of change needed to routines; no relevant data were identified for capacity to innovate or for the nature of organizational adoption or funding decisions.

#### Capacity to Innovate

No eligible study directly examined an organization’s systemic capacity to innovate, such as its ability to absorb repeated technological change, maintain innovation infrastructure, or learn across implementation cycles.

#### Readiness for This Technology

Organizational readiness was constrained by fragmented implementation processes, poor resource allocation, and limited integration with clinical information systems [37,38]. An analysis of user reviews found that 39 of 46 stroke apps had been developed by nonmedical or noneducational entities, often with limited clinical validation or alignment with health-system standards [40]. A patient noted, *“*With the stroke, once you actually leave hospital, that’s it. That’s all. I’ve had absolutely no follow-up, none whatsoever...They, they just toss you out the door, and that’s it; you’re on your own*”* [20]. Facilitators included leadership support, protected implementation time, clinical validation, staff training, and clearly assigned responsibility for monitoring and follow-up.

#### Nature of the Adoption or Funding Decision

No eligible study directly evaluated how organizations made formal adoption, procurement, or internal funding decisions. References to reimbursement and public funding were coded under the wider-system domain because they concerned policy-level arrangements rather than a documented organizational decision-making process.

#### Extent of Change Needed to Routines

Because stroke care is multidisciplinary and crosses organizational boundaries, DHT implementation may require substantial changes to routines [18]. Studies identified a need for standardized digital care pathways, validated repositories, and mechanisms for sharing information across settings [30]. Dedicated implementation coordinators could support interoperability, training, and communication between technology teams and clinical services [36]. Caregivers should be recognized as optional partners with defined roles and support, not as substitutes for professional care [38]. One HCP said, *“*I believe that non-governmental organizations and hospitals can work together to help stroke patients, primarily to involve those responsible*”* [37]. Technologies that did not align with person-centered, integrated care risked superficial use or abandonment [44]. Organizational readiness depends on favorable conditions, such as leadership support, clear roles, and protected time for adaptation [65].

### Wider System Domain

The wider system domain encompasses political, economic, and regulatory contexts that shape the implementation of DHTs and influence their scalability and long-term sustainability. Only 6 included studies reported determinants operating at the broader health care, social, or policy system level.

#### Political/policy Context

Despite the proliferation of DHTs, evidence to guide policy remained limited [35,65]. While technology-supported models may succeed temporarily through local improvisations or pilot projects, they ultimately falter without supportive regulatory frameworks and sustainable financing [38]. Long-term viability demands aligned policy, standardized protocols, and systemic investment, not just technical innovation [40].

#### Regulatory/legal Issues

While participants acknowledged that data sharing enables personalized feedback, it was also perceived as requiring trade-offs in personal privacy. As a clinical manager noted, “when you want to understand somebody, data must be collected in several areas, and based on these data you can provide feedback. So, you give up some privacy to be coached" [28]. The use of cameras or images within apps raised additional privacy concerns for some users [56]. Another requirement involves obtaining patient consent for sharing sensitive data [38]. The issue is the authenticity of the app, as users felt they were being scammed into paying for features within the app [35]. Moreover, users who contacted customer care services for assistance did not receive a reply, which increased their fears [40]. Unclear reimbursement and payment arrangements created uncertainty for providers and could exclude people unable or unwilling to pay. Trust therefore depended on transparent data practices, credible governance, responsive support, and equitable financing [56].

#### Professional Context

No eligible study directly examined how professional regulation, accreditation, liability standards, or interprofessional jurisdiction shaped implementation.

#### Sociocultural Context at the Wider-System Level

No eligible study directly examined population-level sociocultural norms or public discourse as wider-system determinants. Patient-level and family-level cultural findings were retained in the sociocultural factors domain.

### Interaction and Adaptation Over Time

No study directly evaluated the seventh NASSS domain. The evidence focused on anticipated use, co-design, cross-sectional experience, or short-term feasibility; it did not track how DHTs were adapted, abandoned, embedded, scaled, spread, or sustained in routine care. This absence limits conclusions about long-term sustainability.

## Discussion

### Principal Findings

In this review, we synthesized findings from 42 studies to examine stakeholder perspectives on DHTs in poststroke care. The findings were organized within the NASSS framework. The DHT-specific subdomains identified may inform the future development and optimization of DHTs [26]. The earliest included study was published in 2019, and the number of publications increased from 2022 onward, reflecting the rapid development of stroke-related DHTs in recent years [4]. Our findings indicate that the 4 stakeholder groups recognize the importance of the proposed functions, features, and user interface design elements of DHTs, such as personalization, feedback mechanisms, and alert functions. These are seen as essential for the effective use of DHTs in stroke care. A key concern raised by stakeholders related to data security, its ownership, and payment for DHTs. Despite the evident benefits that DHTs could offer patients with stroke in terms of their health, patients emphasized that safety and reliability must be guaranteed as a prerequisite for use. In particular, the supply side adopts a supportive role, focusing on aligning technological solutions with user needs. Our review also revealed that there is limited discussion of clinical and technical accountability among regulators and policymakers. Evidence concerning the adoption of regulatory standards was limited [67]. This suggests that further research should focus on regulatory aspects, particularly in the context of clinical implementation [14].

### Personalization, Usability, and Feedback

The perceived usefulness and ease of use of technology are the most significant determinants of its adoption, providing users with a sense of agency in managing their stroke condition [40]. Although designing tailored DHTs is more time-consuming than adopting a one-size-fits-all approach, this approach enables consideration of the unique characteristics, needs, and preferences of different stroke populations, which may enhance user experience, engagement, and adherence [18,32]. Therefore, tailoring both functionality and interface design to specific stroke subgroups is essential [35]. Patients emphasized the need for design elements that favor simplicity and are easy to use and intuitive. For older adults in particular, providing technical support tailored to their needs can enhance their digital skills and address usability barriers [68]. The number of tailored versions required depends on the nature of the condition, associated comorbidities, and sociocultural factors related to stroke. Moreover, by integrating wearable DHTs into clinical care, personalized, data-driven intervention effects can lead to results that are superior to those achieved with the use of generalized clinical data [20]. DHTs can be used to provide personalized health recommendations based on specific patient data, which has the potential to motivate patients, modify their behavior, and tailor their medication and treatment plans [20,69]. Therefore, features of DHTs should facilitate a personalized approach to meet patients’ needs. The findings highlight the critical role of timely and relevant feedback within DHTs in supporting stroke survivors’ engagement and facilitating the monitoring of disease progression. Both patients and clinicians value personalized, frequent feedback mechanisms, as these help to sustain motivation and enhance recovery tracking [29,59]. Patient portals have the potential to enhance patient engagement in managing their health by allowing access, for example, to discharge summaries, medications, laboratory results, and secure patient-provider communication. Providing visual, auditory, automated, and real-time feedback enhances the user experience, promotes emotional resilience, and supports long-term recovery [70].

### Digital Literacy and Human Support

To reduce the “digital divide,” ongoing education and programs are essential to enhance digital literacy among both patients and HCPs, ensuring they can effectively use DHTs [19]. DHTs can be delivered either as a purely self-help tool or with human guidance, and incorporating human guidance has been shown to enhance treatment adherence and improve outcomes. In addition, software developers should involve end users in the design and testing phases to ensure that DHT systems align with users’ contextual needs and seamlessly integrate into existing routines. Empowering patients with access to their health data not only fosters a sense of control over their own health management but also enhances patient satisfaction [38]. This active involvement can strengthen the patient-provider relationship by promoting shared decision-making and encouraging greater engagement in the management of their condition. The success of such use is often dependent on coordination across home, clinic, and rehabilitation center networks. The role of technology is to facilitate this, rather than to create fragmentation. Caregivers can be better supported through structured training programs, such as toolkits, workshops, or digital courses that build their competence in using DHTs and understanding stroke-related needs [37,46]. Additionally, integrating peer support groups can reduce isolation and foster resilience. Ensuring caregivers have ongoing access to professional guidance and emotional support empowers them to provide consistent, person-centered care [71].

### Financing, Regulation, and Data Privacy

Consistent with previous systematic reviews, this study identified a scarcity of health economic evaluations of DHTs in stroke care [72]. There remains a recognized need for ongoing regulation to govern the clinical use of DHTs and the contexts in which they are implemented, underscoring the importance of further research into regulatory frameworks, particularly regarding clinical integration [14]. National-level research funding accounted for the largest proportion of support for the included studies, highlighting its critical role in driving high-quality research on DHTs in stroke care. To ensure equitable access, policy-level decisions are required on reimbursement, welfare support, and public investment, with the aim of positioning digital health as a public good rather than a consumer product. Safety and confidentiality are important ethical issues in DHTs. Studies suggest that users often hesitate to share their personal information with unfamiliar suppliers due to fear of data misuse. Consequently, this affects how users interact with the technology [61]. The amount of personal and health data collected varies depending on the purpose of the intervention; therefore, it is essential to inform users about what data are collected, how they are used, and where they are stored [12].

### Implications for Practice

To our knowledge, this is the first systematic review–level synthesis of stroke-related DHT evidence organized using the NASSS framework. This review summarizes the key priorities for optimizing DHTs. The general positive attitude toward DHTs among stakeholders creates a foundation of trust [4]. To maximize impact, future practice should prioritize delivering specific, personalized information and interventions tailored to the diverse characteristics and preferences of patients with stroke. The involvement of family members in the implementation and management of DHTs is important to enhance social and mental support. To ensure the effectiveness and quality of management programs, it is essential for health care providers and other program stakeholders to strengthen training and education, clearly define responsibilities, establish communication platforms, and create supervision and feedback processes [67]. Moreover, given the identified resource limitations, future research should better define the cost-effectiveness and equitable distribution of stroke-related DHTs to ensure long-term sustainability.

### Recommendations and Future Directions

Future implementation of DHTs in poststroke care should be treated as a system-level program jointly owned by patients, caregivers, HCPs, developers, organizations, and policymakers. From the outset, implementation plans should define clinical ownership, patient and caregiver support, staff training, workflow integration, escalation pathways, technical maintenance, interoperability, data governance, and sustainable financing. Particular attention should be given to equitable access and to accommodating stroke-related motor, cognitive, communication, and sensory limitations [38]. The absence of evidence regarding interaction and adaptation over time in the NASSS framework also highlights a critical research priority. Longitudinal mixed methods studies embedded in routine care should combine repeated stakeholder assessments with usage, clinical, organizational, and economic data to examine adaptation, scale-up, and sustainability across settings and service transitions. Consistent reporting of implementation processes and contextual changes would help distinguish initial acceptability from sustained adoption and identify which support mechanisms remain effective over time.

### Limitations

Several limitations should be acknowledged. First, restricting the review to English-language, peer-reviewed publications and excluding gray literature, conference proceedings, and trial registries may have introduced language and publication bias. Second, although the search combined controlled vocabulary and free-text terms, the diversity and rapidly evolving terminology of DHTs may have led to the omission of relevant terms or studies. Third, interrater agreement coefficients were not prospectively calculated or retained for study screening, quality appraisal, or NASSS mapping. Fourth, the evidence primarily represented patients, caregivers, and HCPs, with limited input from software developers, industry representatives, and policymakers. This restricted consideration of commercialization, regulation, and scalability. Finally, substantial heterogeneity in DHT functions, apps, settings, and target populations may have contributed to variability across findings and limited their generalizability.

## Conclusions

Successful implementation of DHTs in poststroke care requires more than technical functionality. Technologies should accommodate stroke-related impairments, digital access and literacy, caregiver capacity, and the routines of multidisciplinary services. Organizations should define clinical ownership, training, interoperability, data governance, and sustainable financing before deployment. NASSS can be used prospectively to identify implementation complexity and evidence gaps, but the present literature provides insufficient longitudinal evidence to infer sustained use, scale-up, or spread. Future studies should therefore evaluate adaptation and outcomes over time while reporting how patient, caregiver, professional, organizational, and policy priorities are addressed.

## Supplementary material

10.2196/95197 Multimedia Appendix 1 Search strategies.

10.2196/95197 Multimedia Appendix 2 Study characteristics.

10.2196/95197 Multimedia Appendix 3 Quality appraisal.

10.2196/95197 Checklist 1 PRISMA checklist.

## Abbreviations

DHT: digital health technology
GBD: Global Burden of Disease
HCP: health care professional
MMAT: Mixed Methods Appraisal Tool
NASSS: Nonadoption, Abandonment, Scale-Up, Spread, and Sustainability
PRISMA: Preferred Reporting Items for Systematic Reviews and Meta-Analyses
PROSPERO: International Prospective Register of Systematic Reviews
VERA: Virtual Engagement Rehabilitation Assistant
