# Supplementary material for: Stakeholder Perspectives on Digital Health Technologies in Poststroke Self-Management and Rehabilitation: Systematic Review Based on the NASSS Framework
Source: JMIR Mhealth Uhealth. 2026 Oct 9;14:e95197. doi: 10.2196/95197 (PMC13656741; doi:10.2196/95197)
Supplement: Supplementary file 1 [file mhealth-v14-e95197-s001.docx]

Stakeholder Perspectives on Digital Health Technologies in Post-Stroke Self-Management and Rehabilitation: A Systematic Review Based on the NASSS Framework

Contents

[Appendix 1. Search strategy and search results	2](#_Toc25042)

[1.1 Embase	2](#_Toc17906)

[1.2 CINAHL	3](#_Toc12220)

[1.3 Scopus	5](#_Toc1802)

[1.4 Web of Science	6](#_Toc32142)

[1.5 MEDLINE	7](#_Toc13676)

[1.6 PubMed	9](#_Toc18816)

**Appendix 1.** Search strategy and search results

|  | Search results October 2025 in full |
| --- | --- |
| PubMed | 2,519 |
| MEDLINE | 2,928 |
| Embase | 53 |
| Scopus | 7,176 |
| EBSCOhost CINAHL | 1,754 |
| Web of Science | 789 |
| Additional records identified through other sources | 43 |
| Total | 15,262 |
| Total after deduplication | 9,770 |

1.1 Embase

Limits：Human subjects, language (English), no reviews/meta-analyses

#1 'stroke':ab,ti,kw OR 'strokes':ab,ti,kw OR 'cerebrovascular accident'/exp OR 'cerebrovascular accidents':ab,ti,kw OR 'cerebrovascular apoplexy':ab,ti,kw OR 'apoplexy, cerebrovascular':ab,ti,kw OR 'brain vascular accident':ab,ti,kw OR 'apoplexy':ab,ti,kw OR 'cva cerebrovascular accident':ab,ti,kw OR 'cerebrovascular accident, acute':ab,ti,kw OR 'acute cerebrovascular accident':ab,ti,kw OR 'brain edema'/exp OR 'intracranial hypertension'/exp OR 'malignant cerebral edema':ab,ti,kw OR 'brain swelling'/exp OR 'large hemispheric infarction':ab,ti,kw OR 'massive cerebral infarction':ab,ti,kw OR 'mca infarction':ab,ti,kw OR 'ischemic stroke'/exp OR 'cerebral infarction':ab,ti,kw OR 'cerebral infarctions':ab,ti,kw OR 'infarctions, cerebral':ab,ti,kw OR 'cerebral infarct':ab,ti,kw OR 'cerebral infarcts':ab,ti,kw OR 'infarct, cerebral':ab,ti,kw OR 'infarcts, cerebral':ab,ti,kw OR 'infarction, cerebral':ab,ti,kw OR 'anterior choroidal artery infarction':ab,ti,kw OR 'posterior choroidal artery infarction':ab,ti,kw OR 'subcortical infarction':ab,ti,kw OR 'accident, cerebrovascular':ab,ti,kw OR 'acute cerebrovascular lesion':ab,ti,kw OR 'acute stroke':ab,ti,kw OR 'acutefocal cerebral vasculopathy':ab,ti,kw OR 'apoplectic stroke':ab,ti,kw OR 'apoplexia':ab,ti,kw OR 'blood flow disturbance, brain':ab,ti,kw OR 'brain accident ':ab,ti,kw OR 'brain blood flow':ab,ti,kw OR 'brain insult':ab,ti,kw OR 'brain insultus':ab,ti,kw OR 'cerebral apoplexia':ab,ti,kw OR 'cerebral insult':ab,ti,kw OR 'cerebral stroke':ab,ti,kw OR 'cerebral vascular accident':ab,ti,kw OR 'cerebralvascular insufficiency':ab,ti,kw OR 'cerebro vascular accident':ab,ti,kw OR 'cerebrovascular arrest':ab,ti,kw OR 'cerebrovascular failure':ab,ti,kw OR 'cerebrovascular injury':ab,ti,kw OR 'cerebrovascular insufficiency':ab,ti,kw OR 'cerebrovascular insult':ab,ti,kw OR 'cerebrum vascular accident':ab,ti,kw OR 'cryptogenic stroke':ab,ti,kw OR 'cva':ab,ti,kw OR 'insultus cerebralis':ab,ti,kw OR 'ischaemic seizure':ab,ti,kw OR 'ischemic seizure':ab,ti,kw OR 'thrombotic stroke':ab,ti,kw OR 'Hemorrhagic Strokes':ab,ti,kw OR 'Hemorrhagic Stroke':ab,ti,kw OR 'Stroke, Hemorrhagic':ab,ti,kw OR 'Stroke, Intracerebral Hemorrhagic':ab,ti,kw OR 'Intracerebral Hemorrhage Stroke':ab,ti,kw OR 'Hemorrhage Stroke, Intracerebral':ab,ti,kw OR 'Intracerebral Hemorrhage Strokes':ab,ti,kw OR 'Stroke, Intracerebral Hemorrhage':ab,ti,kw OR 'Stroke, Subarachnoid Hemorrhagic':ab,ti,kw OR 'Cerebral Hemorrhage':ab,ti,kw OR 'Hemorrhage, Cerebral':ab,ti,kw OR 'Cerebral Hemorrhages':ab,ti,kw OR 'Hemorrhages, Cerebral':ab,ti,kw OR 'Intracerebral Hemorrhage':ab,ti,kw OR 'Hemorrhage, Intracerebral':ab,ti,kw OR 'Hemorrhages, Intracerebral':ab,ti,kw OR 'Intracerebral Hemorrhages':ab,ti,kw OR 'Hemorrhage, Cerebrum':ab,ti,kw OR 'Cerebrum Hemorrhage':ab,ti,kw OR 'Cerebrum Hemorrhages':ab,ti,kw OR 'Cerebral Brain Hemorrhages':ab,ti,kw OR 'Hemorrhage, Cerebral Brain':ab,ti,kw OR 'Cerebral Parenchymal Hemorrhage':ab,ti,kw OR 'Hemorrhage, Cerebral Parenchymal':ab,ti,kw OR 'Hemorrhages, Cerebral Parenchymal':ab,ti,kw OR 'Putaminal Hemorrhage ':ab,ti,kw OR 'Brain Hemorrhages, Putaminal':ab,ti,kw OR 'Putamen Hemorrhage':ab,ti,kw OR 'Putaminal Hematoma':ab,ti,kw OR 'Intracranial Hemorrhage':ab,ti,kw OR 'Hemorrhages, Intracranial':ab,ti,kw OR 'Hemorrhage, Intracranial':ab,ti,kw OR 'Brain Hemorrhage':ab,ti,kw OR 'Hemorrhage, Brain':ab,ti,kw OR 'Hemorrhages, Brain':ab,ti,kw OR 'Posterior Fossa Hemorrhage':ab,ti,kw OR 'Hemorrhage, Posterior Fossa':ab,ti,kw OR 'Hemorrhages, Posterior Fossa':ab,ti,kw

854,562

#2 'digital health'/exp OR 'digital healthor digital health technology':ab,ti OR 'health, digital':ab,ti OR 'digital health technologies':ab,ti OR 'health technologies, digital':ab,ti OR 'health technology, digital':ab,ti

6965

#3 opinions:ab,ti OR perceptions:ab,ti OR view:ab,ti OR experience:ab,ti OR attitudes:ab,ti OR qualitative:ab,ti OR perspective*:ab,ti OR 'case stud*':ab,ti OR interview*:ab,ti OR 'focus group*':ab,ti OR 'participant observation':ab,ti

3,603,190

#4 #1 AND #2 AND #3

**Search Results: 53**

1.2 CINAHL

#1 MH "Hemorrhagic Stroke" OR MH "Subarachnoid Hemorrhage" OR MH "Intracranial Hemorrhage" OR MH "Cerebral Hemorrhage" OR MH"Stroke+" OR XB Strokes OR XB Cerebrovascular Accident OR XB Cerebrovascular Apoplexy OR XB Vascular Accident, Brain OR XB Brain Vascular Accident OR XB Apoplexy OR XB Brain Edema OR MH"Intracranial Hypertension+" OR XB malignant cerebral edema OR XB brain swelling OR XB large hemispheric infarction OR XB massive cerebral infarction OR XB MCA infarction OR MH"Ischemic Stroke+" OR MH"Cerebral Infarction+" OR XB Cerebral Infarctions OR XB Infarctions, Cerebral OR XB Cerebral Infarct OR XB Anterior Choroidal Artery Infarction OR XB Subcortical Infarction OR XB Infarctions, Subcortical OR XB Cerebral Edema OR XB cerebral arterial thrombosis OR XB Ischemic Cerebrovascular Disease OR XB Intracranial Thrombosis OR XB Intracranial Thromboses OR XB Thromboses, Intracranial OR XB Thrombosis, Intracranial OR XB Thrombus, Intracranial OR XB Intracranial Thrombus OR XB Cerebral Thrombosis OR XB Brain Thrombosis OR XB Cerebral Thrombus OR XB Thrombus, Cerebral OR MH"Intracranial Embolism+" OR XB Embolism, Intracranial OR XB Cerebral Embolism OR XB Embolism, Cerebral OR XB Cerebral Emboli OR XB Cerebral Embolus OR XB Emboli, Cerebral OR XB Embolus, Cerebral OR XB Brain Embolism OR XB Brain Emboli OR XB Brain Embolus OR XB Emboli, Brain OR XB Embolus, Brain OR XB Embolism, Brain OR XB acute ischemic stroke OR XB acute ischemic strokes OR XB Stroke, Acute OR XB Acute Strokes OR XB Acute Thrombotic Stroke OR XB cva cerebrovascular accident OR XB brain infarction OR XB accident, cerebrovascular OR XB acute cerebrovascular lesion OR XB acute stroke OR XB acutefocal cerebral vasculopathy OR XB apoplectic stroke OR XB apoplexia OR XB blood flow disturbance, brain OR XB brain accident OR XB brain blood flow OR XB brain insult OR XB brain insultus OR XB cerebral apoplexia OR XB cerebral insult OR XB cerebral stroke OR XB cerebral vascular accident OR XB cerebralvascular insufficiency OR XB cerebro vascular accident OR XB cerebrovascular arrest OR XB cerebrovascular failure OR XB cerebrovascular injury OR XB cerebrovascular insufficiency OR XB cerebrovascular insult OR XB cerebrum vascular accident OR XB cryptogenic stroke OR XB cva OR XB insultus cerebralis OR XB ischaemic seizure OR XB ischemic seizure OR XB thrombotic stroke OR XB Hemorrhagic Strokes OR XB Hemorrhagic Stroke OR XB Stroke, Hemorrhagic OR XB Stroke, Intracerebral Hemorrhagic OR XB Intracerebral Hemorrhage Stroke OR XB Hemorrhage Stroke, Intracerebral OR XB Intracerebral Hemorrhage Strokes OR XB Stroke, Intracerebral Hemorrhage OR XB Stroke, Subarachnoid Hemorrhagic OR XB Cerebral Hemorrhage OR XB Hemorrhage, Cerebral OR XB Cerebral Hemorrhages OR XB Hemorrhages, Cerebral OR XB Intracerebral Hemorrhage OR XB Hemorrhage, Intracerebral OR XB Hemorrhages, Intracerebral OR XB Intracerebral Hemorrhages OR XB Hemorrhage, Cerebrum OR XB Cerebrum Hemorrhage OR XB Cerebrum Hemorrhages OR XB Cerebral Brain Hemorrhages OR XB Hemorrhage, Cerebral Brain OR XB Cerebral Parenchymal Hemorrhage OR XB Hemorrhage, Cerebral Parenchymal OR XB Hemorrhages, Cerebral Parenchymal OR XB Putaminal Hemorrhage OR XB Brain Hemorrhages, Putaminal OR XB Putamen Hemorrhage OR XB Putaminal Hematoma OR XB Intracranial Hemorrhage OR XB Hemorrhages, Intracranial OR XB Hemorrhage, Intracranial OR XB Brain Hemorrhage OR XB Hemorrhage, Brain OR XB Hemorrhages, Brain OR XB Posterior Fossa Hemorrhage OR XB Hemorrhage, Posterior Fossa OR XB Hemorrhages, Posterior Fossa

344,708

#2 MH "Digital Health+" OR MH "Digital Technology+" OR MH "Wearable Sensors+" OR MH "Mobile Applications+" OR XB digital health technologies OR XB digital health technology OR XB digital health OR XB Health Technology OR XB mobile health OR XB digital treatment OR XB e-health OR XB ehealth OR XB smartwatch OR XB health app OR XB app OR XB application OR XB SMS OR XB digital medicine OR XB Digital Technology OR XB Wearable Sensors OR XB Mobile Applications

546,136

#3 MH "Perception" OR MH "Attitude" OR MH "Interviews" OR XB opinion OR XB opinions OR XB perception OR XB perceptions OR XB view OR XB experience OR XB experiences OR XB attitude OR XB attitudes OR XB Qualitative OR XB Perspective OR XB Perspectives OR XB interview OR XB interviews OR XB quantitative OR XB mixed-method OR XB mixed-methods

2,450,316

#4 #1 AND #2 AND #3

**Search Results: 1,754**

1.3 Scopus

TITLE-ABS-KEY (“Strokes” or “Cerebrovascular Accident” or “Cerebrovascular Apoplexy” or “Vascular Accident, Brain” or “Brain Vascular Accident” or “Apoplexy” or “Brain Edema” or “malignant cerebral edema” or “brain swelling” or “large hemispheric infarction” or “massive cerebral infarction” or “MCA infarction” or “Cerebral Infarctions” or “Infarctions, Cerebral” or “Cerebral Infarct” or “Anterior Choroidal Artery Infarction” or “Subcortical Infarction” or “Infarctions, Subcortical” or “Cerebral Edema” or “cerebral arterial thrombosis” or “Ischemic Cerebrovascular Disease” or “Intracranial Thrombosis” or “Intracranial Thromboses” or “Thromboses, Intracranial” or “Thrombosis, Intracranial” or “Thrombus, Intracranial” or “Intracranial Thrombus” or “Cerebral Thrombosis” or “Brain Thrombosis” or “Cerebral Thrombus” or “Thrombus, Cerebral” or “Embolism, Intracranial” or “Cerebral Embolism” or “Embolism, Cerebral” or “Cerebral Emboli” or “Cerebral Embolus” or “Emboli, Cerebral” or “Embolus, Cerebral” or “Brain Embolism” or “Brain Emboli” or “Brain Embolus” or “Emboli, Brain” or “Embolus, Brain” or “Embolism, Brain” or “acute ischemic stroke” or “acute ischemic strokes” or “Stroke, Acute” or “Acute Strokes” or “Acute Thrombotic Stroke” or “cva cerebrovascular accident” or “brain infarction” or “accident, cerebrovascular” or “acute cerebrovascular lesion” or “acute stroke” or “acutefocal cerebral vasculopathy” or “apoplectic stroke” or “apoplexia” or “blood flow disturbance, brain” or “brain accident” or “brain blood flow” or “brain insult” or “brain insultus” or “cerebral apoplexia” or “cerebral insult” or “cerebral stroke” or “cerebral vascular accident” or “cerebralvascular insufficiency” or “cerebro vascular accident” or “cerebrovascular arrest” or “cerebrovascular failure” or “cerebrovascular injury” or “cerebrovascular insufficiency” or “cerebrovascular insult” or “cerebrum vascular accident” or “cryptogenic stroke” or “cva” or “insultus cerebralis” or “ischaemic seizure” or “ischemic seizure” or “thrombotic stroke” or “Hemorrhagic Strokes” or “Hemorrhagic Stroke” or “Stroke, Hemorrhagic” or “Stroke, Intracerebral Hemorrhagic” or “Intracerebral Hemorrhage Stroke” or “Hemorrhage Stroke, Intracerebral” or “Intracerebral Hemorrhage Strokes” or “Stroke, Intracerebral Hemorrhage” or “Stroke, Subarachnoid Hemorrhagic” or “Cerebral Hemorrhage” or “Hemorrhage, Cerebral” or “Cerebral Hemorrhages” or “Hemorrhages, Cerebral” or “Intracerebral Hemorrhage” or “Hemorrhage, Intracerebral” or “Hemorrhages, Intracerebral” or “Intracerebral Hemorrhages” or “Hemorrhage, Cerebrum” or “Cerebrum Hemorrhage” or “Cerebrum Hemorrhages” or “Cerebral Brain Hemorrhages” or “Hemorrhage, Cerebral Brain” or “Cerebral Parenchymal Hemorrhage” or “Hemorrhage, Cerebral Parenchymal” or “Hemorrhages, Cerebral Parenchymal” or “Putaminal Hemorrhage” or “Brain Hemorrhages, Putaminal” or “Putamen Hemorrhage” or “Putaminal Hematoma” or “Intracranial Hemorrhage” or “Hemorrhages, Intracranial” or “Hemorrhage, Intracranial” or “Brain Hemorrhage” or “Hemorrhage, Brain” or “Hemorrhages, Brain” or “Posterior Fossa Hemorrhage” or “Hemorrhage, Posterior Fossa” or “Hemorrhages, Posterior Fossa”)

909,317

TITLE-ABS-KEY (“digital health technologies” or “digital health technology” or “digital health” or “Health Technology” or “mobile health” or “digital treatment” or “e-health” or “ehealth” or “smartwatch” or “health app” or “app” or “application” or “SMS” or “digital medicine” or “Digital Technology” or “Wearable Sensors” or “Mobile Applications”)

9,648,024

TITLE-ABS-KEY (“opinion” or “opinions” or “perception” or “perceptions” or “view” or “experience” or “experiences” or “attitude” or “attitudes” or “Qualitative” or “Perspective” or “Perspectives” or “interview” or “interviews” or “quantitative” or “mixed-method” or “mixed-methods”)

11,297,485

#4 #1 AND #2 AND #3

**Search Results: 7,176**

1.4 Web of Science

#1 TS=(Stroke OR Cerebrovascular Accident OR Cerebrovascular Apoplexy OR Vascular Accident, Brain OR Apoplexy OR Brain Edema OR Intracranial Hypertension OR malignant cerebral edema OR brain swelling OR large hemispheric infarction OR massive cerebral infarction OR MCA infarction OR Cerebral Infarction OR Cerebral Infarct OR Anterior Choroidal Artery Infarction OR Posterior Choroidal Artery Infarction OR Subcortical Infarction OR Cerebral Edema OR cerebral arterial thrombosis OR Ischemic Cerebrovascular Disease OR Intracranial Thrombosis OR Thrombus, Intracranial OR Cerebral Thrombosis Cerebral Thrombus OR Intracranial Embolism OR Cerebral Embolism OR Embolism, Cerebral OR Cerebral Emboli OR Brain Embolism OR Brain Emboli OR accident, cerebrovascular OR acute cerebrovascular lesion OR acute stroke OR acutefocal cerebral vasculopathy OR apoplectic stroke OR apoplexia OR blood flow disturbance, brain OR brain accident OR brain blood flow OR brain insult OR brain insultus OR cerebral apoplexia OR cerebral insult OR cerebral stroke OR cerebral vascular accident OR cerebralvascular insufficiency OR cerebro vascular accident OR cerebrovascular arrest OR cerebrovascular failure OR cerebrovascular injury OR cerebrovascular insufficiency OR cerebrovascular insult OR cerebrum vascular accident OR cryptogenic stroke OR cva OR insultus cerebralis OR ischaemic seizure OR ischemic seizure OR thrombotic stroke OR Hemorrhagic Strokes OR Hemorrhagic Stroke OR Stroke, Hemorrhagic OR Stroke, Intracerebral Hemorrhagic OR Intracerebral Hemorrhage Stroke OR Hemorrhage Stroke, Intracerebral OR Intracerebral Hemorrhage Strokes OR Stroke, Intracerebral Hemorrhage OR Stroke, Subarachnoid Hemorrhagic OR Cerebral Hemorrhage OR Hemorrhage, Cerebral OR Cerebral Hemorrhages OR Hemorrhages, Cerebral OR Intracerebral Hemorrhage OR Hemorrhage, Intracerebral OR Hemorrhages, Intracerebral OR Intracerebral Hemorrhages OR Hemorrhage, Cerebrum OR Cerebrum Hemorrhage OR Cerebrum Hemorrhages OR Cerebral Brain Hemorrhages OR Hemorrhage, Cerebral Brain OR Cerebral Parenchymal Hemorrhage OR Hemorrhage, Cerebral Parenchymal OR Hemorrhages, Cerebral Parenchymal OR Putaminal Hemorrhage OR Brain Hemorrhages, Putaminal OR Putamen Hemorrhage OR Putaminal Hematoma OR Intracranial Hemorrhage OR Hemorrhages, Intracranial OR Hemorrhage, Intracranial OR Brain Hemorrhage OR Hemorrhage, Brain OR Hemorrhages, Brain OR Posterior Fossa Hemorrhage OR Hemorrhage, Posterior Fossa OR Hemorrhages, Posterior Fossa)

402,688

#2 (TS=(digital health OR digital health technology OR health, digital OR digital health technologies OR health technologies OR health technology OR digital treatment* OR mHealth OR application OR mobile health OR app*)

87,549

#3 (TS=(opinion* OR perception* OR view* OR experience* OR attitude* OR qualitative OR perspective OR case study OR interview OR behavior* OR preference* OR determinants OR barrier OR thoughts OR belief OR focus group OR participant observation)

5,541,747

#4 #1 and #2 and #3

**Search Results: 789**

1.5 MEDLINE

#1 MH "Hemorrhagic Stroke" OR MH "Subarachnoid Hemorrhage" OR MH "Intracranial Hemorrhage" OR MH "Cerebral Hemorrhage" OR MH"Stroke+" OR XB Strokes OR XB Cerebrovascular Accident OR XB Cerebrovascular Apoplexy OR XB Vascular Accident, Brain OR XB Brain Vascular Accident OR XB Apoplexy OR XB Brain Edema OR MH"Intracranial Hypertension+" OR XB malignant cerebral edema OR XB brain swelling OR XB large hemispheric infarction OR XB massive cerebral infarction OR XB MCA infarction OR MH"Ischemic Stroke+" OR MH"Cerebral Infarction+" OR XB Cerebral Infarctions OR XB Infarctions, Cerebral OR XB Cerebral Infarct OR XB Anterior Choroidal Artery Infarction OR XB Subcortical Infarction OR XB Infarctions, Subcortical OR XB Cerebral Edema OR XB cerebral arterial thrombosis OR XB Ischemic Cerebrovascular Disease OR XB Intracranial Thrombosis OR XB Intracranial Thromboses OR XB Thromboses, Intracranial OR XB Thrombosis, Intracranial OR XB Thrombus, Intracranial OR XB Intracranial Thrombus OR XB Cerebral Thrombosis OR XB Brain Thrombosis OR XB Cerebral Thrombus OR XB Thrombus, Cerebral OR MH"Intracranial Embolism+" OR XB Embolism, Intracranial OR XB Cerebral Embolism OR XB Embolism, Cerebral OR XB Cerebral Emboli OR XB Cerebral Embolus OR XB Emboli, Cerebral OR XB Embolus, Cerebral OR XB Brain Embolism OR XB Brain Emboli OR XB Brain Embolus OR XB Emboli, Brain OR XB Embolus, Brain OR XB Embolism, Brain OR XB acute ischemic stroke OR XB acute ischemic strokes OR XB Stroke, Acute OR XB Acute Strokes OR XB Acute Thrombotic Stroke OR XB cva cerebrovascular accident OR XB brain infarction OR XB accident, cerebrovascular OR XB acute cerebrovascular lesion OR XB acute stroke OR XB acutefocal cerebral vasculopathy OR XB apoplectic stroke OR XB apoplexia OR XB blood flow disturbance, brain OR XB brain accident OR XB brain blood flow OR XB brain insult OR XB brain insultus OR XB cerebral apoplexia OR XB cerebral insult OR XB cerebral stroke OR XB cerebral vascular accident OR XB cerebralvascular insufficiency OR XB cerebro vascular accident OR XB cerebrovascular arrest OR XB cerebrovascular failure OR XB cerebrovascular injury OR XB cerebrovascular insufficiency OR XB cerebrovascular insult OR XB cerebrum vascular accident OR XB cryptogenic stroke OR XB cva OR XB insultus cerebralis OR XB ischaemic seizure OR XB ischemic seizure OR XB thrombotic stroke OR XB Hemorrhagic Strokes OR XB Hemorrhagic Stroke OR XB Stroke, Hemorrhagic OR XB Stroke, Intracerebral Hemorrhagic OR XB Intracerebral Hemorrhage Stroke OR XB Hemorrhage Stroke, Intracerebral OR XB Intracerebral Hemorrhage Strokes OR XB Stroke, Intracerebral Hemorrhage OR XB Stroke, Subarachnoid Hemorrhagic OR XB Cerebral Hemorrhage OR XB Hemorrhage, Cerebral OR XB Cerebral Hemorrhages OR XB Hemorrhages, Cerebral OR XB Intracerebral Hemorrhage OR XB Hemorrhage, Intracerebral OR XB Hemorrhages, Intracerebral OR XB Intracerebral Hemorrhages OR XB Hemorrhage, Cerebrum OR XB Cerebrum Hemorrhage OR XB Cerebrum Hemorrhages OR XB Cerebral Brain Hemorrhages OR XB Hemorrhage, Cerebral Brain OR XB Cerebral Parenchymal Hemorrhage OR XB Hemorrhage, Cerebral Parenchymal OR XB Hemorrhages, Cerebral Parenchymal OR XB Putaminal Hemorrhage OR XB Brain Hemorrhages, Putaminal OR XB Putamen Hemorrhage OR XB Putaminal Hematoma OR XB Intracranial Hemorrhage OR XB Hemorrhages, Intracranial OR XB Hemorrhage, Intracranial OR XB Brain Hemorrhage OR XB Hemorrhage, Brain OR XB Hemorrhages, Brain OR XB Posterior Fossa Hemorrhage OR XB Hemorrhage, Posterior Fossa OR XB Hemorrhages, Posterior Fossa

511,592

#2 MH "Digital Health+" OR MH "Digital Technology+" OR MH "Wearable Sensors+" OR MH "Mobile Applications+" OR XB digital health technologies OR XB digital health technology OR XB digital health OR XB Health Technology OR XB mobile health OR XB digital treatment OR XB e-health OR XB ehealth OR XB smartwatch OR XB health app OR XB app OR XB application OR XB SMS OR XB digital medicine OR XB Digital Technology OR XB Wearable Sensors OR XB Mobile Applications

2,013,901

#3 MH "Perception" OR MH "Attitude" OR MH "Interviews" OR XB opinion OR XB opinions OR XB perception OR XB perceptions OR XB view OR XB experience OR XB experiences OR XB attitude OR XB attitudes OR XB Qualitative OR XB Perspective OR XB Perspectives OR XB interview OR XB interviews OR XB quantitative OR XB mixed-method OR XB mixed-methods

3,691,225

#4 #1 AND #2 AND #3

**Search Results: 2928**

1.6 PubMed

#1"stroke"[Mesh] OR stroke[Title/Abstract] OR strokes[Title/Abstract] OR "post stroke"[Title/Abstract]OR "post-stroke"[Title/Abstract] OR "poststroke"[Title/Abstract] OR "cerebrovascular accident"[Title/Abstract] OR "cerebrovascular disorder"[Title/Abstract] OR "ischemic stroke"[Title/Abstract] OR “hemorrhagic stroke”[Title/Abstract] OR "CVA" [Title/Abstract] OR "CVAs"[Title/Abstract] OR "cerebrovascular apoplexy"[Title/Abstract] OR “apoplexy”[Title/Abstract] OR "intracerebral hemorrhage" [Title/Abstract] OR "cerebral hemorrhage"[Title/Abstract] OR "cerebral infarction"[Title/Abstract] OR "brain infarction"[Title/Abstract] OR "brain ischemia"[Title/Abstract] OR "cerebrovascular disease"[Title/Abstract] OR "cerebrovascular disorders"[Title/Abstract] OR "brain stem infarctions "[Title/Abstract] OR "cerebral infarction "[Title/Abstract]OR "lacunar infarction"[Title/Abstract]

459,162

#2"digital health"[Mesh] OR digital health[Title/Abstract] OR digital health technologies[Title/Abstract] OR digital health technology[Title/Abstract] OR Health Technology[Title/Abstract] OR mobile health[Title/Abstract] OR digital treatment[Title/Abstract] OR e-health[Title/Abstract] OR ehealth[Title/Abstract] OR smartwatch[Title/Abstract] OR health app[Title/Abstract] OR app[Title/Abstract] OR application[Title/Abstract] OR SMS[Title/Abstract] OR digital medicine[Title/Abstract]

1,306,753

#3"Perception"[Mesh] OR "Attitude"[Mesh] OR opinion[Title/Abstract] OR opinions[Title/Abstract] OR perception[Title/Abstract] OR perceptions[Title/Abstract] OR view[Title/Abstract] OR experience[Title/Abstract] OR experiences[Title/Abstract] OR attitude[Title/Abstract] OR attitudes[Title/Abstract] OR Qualitative[Title/Abstract] OR Perspective[Title/Abstract] OR Perspectives[Title/Abstract] OR interview[Title/Abstract] OR interviews[Title/Abstract] OR quantitative[Title/Abstract] OR mixed-method[Title/Abstract] OR mixed-methods[Title/Abstract]

4,434,743

#4 #1 AND #2 AND #3

**Search Results: 2,519**
