# Supplementary material for: Stakeholder Perspectives on Digital Health Technologies in Poststroke Self-Management and Rehabilitation: Systematic Review Based on the NASSS Framework
Source: JMIR Mhealth Uhealth. 2026 Oct 9;14:e95197. doi: 10.2196/95197 (PMC13656741; doi:10.2196/95197)
Supplement: Supplementary file 2 [file mhealth-v14-e95197-s002.docx]

Stakeholder Perspectives on Digital Health Technologies in Post-Stroke Self-Management and Rehabilitation: A Systematic Review Based on the NASSS Framework

Contents

[Appendix 2. Data extraction table of included studies	2](#_Toc5107)

[2.1.	Qualitative studies（24）	2](#_Toc18552)

[2.2.	Quantitative studies（8）	13](#_Toc5419)

[2.3.	Mixed-methods studies（10）	20](#_Toc26751)

**Appendix 2.** Data extraction table of included studies

2.1.	Qualitative studies（24）

| **Title** | **Author，year** | **Country** | **Aim** | **Target population** | **Participants** | **Data collection and analysis** | **Setting** | **DHT(type )** | **Funding** |
| --- | --- | --- | --- | --- | --- | --- | --- | --- | --- |
|  |  |  |  |  | **Sample size、female，age、characteristics** |  |  |  |  |
| 1.Patients’ perspectives regarding digital health technology to support self-management and improve integrated stroke care: Qualitative interview study | Bally, 2023 | Netherlands | To explore the perspectives of stroke patients toward how digital health technology could support self-management regarding health and well-being, as well as integrated stroke care. | Patients with ischemic stroke | N=36, 15 women and 21 men, 54-90 years old, ischemic stroke within the past 18 months | One-to-one telephone interviews, a thematic approach. | Patients were purposively sampled from a single-site, large, academic hospital in rotterdam, the netherlands. | It includes a wide range of digital technologies for health such as information and communication technology, mobile wireless technology, artificial intelligence, big data, and robotics. | European Commission |
| 2.Technologies to improve the participation of stroke patients in their home environment | Esther m. G. Willems, 2022 | Netherlands | To identify possible technological solutions that can contribute to stroke patients’ participation at home. | Patients with stroke | N=10, Stroke patients (n = 6), 4 men, 2women, and experts (n = 4) | Semi-structured individual interviews, a group interview  "Open, axial and selective coding | The study was carried out among people discharged from a rehabilitation clinic in the limburg and noord-brabant regions of the netherlands. | Digital assistants, apps, and virtual reality were frequently mentioned | No funding |
| 3.The online health information–seeking behaviors of people who have experienced stroke: Qualitative interview study | Brigid clancy，2024 | Australia | To explore the online health information–seeking behaviors of people who have experienced stroke and any related barriers or navigational needs. | Patients with stroke | N=15, 9 women and 6 men,the age of the participants ranged between 28 and 79 years,experienced stroke 6 to 36 months ago | Semistructured one-on-one interviews ,a thematic approach,a combined inductive–deductive approach | The participants resided in 4 australian states | Internet-based resources/online health information | University of Newcastle |
| 4.Exploring people with stroke’s perceptions of digital technologies in post-stroke rehabilitation – a qualitative study | Ann marie hestetun-mandrup,2025 | Norway | To explore how people with stroke perceive the usage of digital technologies in post-stroke rehabilitation. | Adults post-stroke attending outpatient follow-up | N=17, 6 women and 11 men,older than 18 years who had experienced a stroke (haemorrhagic stroke or ischaemic stroke) | Individual semi-structured interviews, reflexive thematic analysis | The interviews were conducted at a rehabilitation hospital in norway | They used various technologies to stay connected to the rehabilitation network, such as video-consultations, apps and exergaming | No funding |
| 5.Perspectives of clients and health care professionals on the opportunities for digital health interventions in cerebrovascular disease care: Qualitative descriptive study | Henna härkönen,2024 | Finland | To identify opportunities for dhis from clients’ and health care professionals’ perspectives to address health system challenges and improve cvd care. | Adults with diagnosed ischemic stroke or tia, and health care professionals directly involved in cvd care | N=48, 22 clients and 26 health care professionals,28women and 20 men | Semistructured individual interviews,deductive and inductive content analysis | A single tertiary-level hospital in finland | It includes mhealth applications, telemedicine, access to electronic medical records, wearables,health information platforms,decision support systems and data exchange and interoperability services | Business Finland |
| 6.A pilot exploration of staﬀ and service-user perceptions of a novel digital health technology (virtual engagement rehabilitation assistant) in complex inpatient rehabilitation | Kathryn jarvis,2025 | UK | To explore staff and service-user perceptions of implementing vera on a uk complex inpatient rehabilitation ward | Inpatients with complex stroke rehabilitation needs and the staff responsible for their care. | N=16, 7 service-users and 9 staff 6 interviewees self-reported as male and one as female.”Staff gender not provided in the text | Semi-structured interviews, using framework analysis | Complex rehabilitation unit (cru) within the uk nhs | Virtual engagement rehabilitation assistant | MedCity’s Collaborate to Innovate in conjunction |
| 7.Exploring the unmet needs of young adults with stroke in australia: Can technology help meet their needs? A qualitative study | Dinah amoah,2023 | Australia | To explore the unmet needs of young adults aged 18–30 years with stroke and ascertain how technology can help meet those needs to improve quality of life and participation | Young adults with stroke, their caregivers, and healthcare professionals | N=16, young adults with stroke (n=10) caregivers of young adults with stroke (n=3) healthcare professionals (n=3) 7 women and 9 men | Semi-structured interviews,reflexive thematic analysis | Participants were residents of various australian states and territories | Technology-based applications | No funding, as a part of a Ph.D. project |
| 8.‘we are very individual’: Anticipated effects on stroke survivors of using their person- generated health data | Gerardo luis dimaguila, 2020 | Australia | To present the perspectives of stroke survivors and clinicians on the anticipated effects of stroke survivors’ use of pghd from a poststroke simulated rehabilitation technology. | Stroke survivors and clinicians (physiotherapists) involved in stroke rehabilitation. | N=11, gender not provided in the text | Semistructured interviews ,deductive thematic analysis | Headway abi (queensland) and a large metropolitan hospital in melbourne (victoria). | The article focuses on person-generated health data (pghd) produced by simulated rehabilitation technologies, specifically kinect-based stroke rehabilitation systems (k-srs) | No funding |
| 9.A qualitative examination of the usability of a digital cognitive behavioral therapy for insomnia program after stroke | Tom smejka,2022 | UK | To explore the experience of people with stroke using the digital cbt-i program “sleepio” And perceptions of usability, and to identify potential barriers to completion. | Community-dwelling survivors of stroke | N=11, 5women and 6men  post-stroke (≥3 months), varied cognitive function (moca scores 10–29), varied sleep quality (sci scores 9–31), some with one-sided weakness, some with cognitive impairments | Semi-structured interviews reflexive thematic analysis | Community-based, post-stroke rehabilitation context. | Digital cognitive behavioral therapy for insomnia (digital cbt-i) | the National  Institute for Health Research (NIHR) Oxford Biomedical Research Centre |
| 10.Optimizing a digital intervention for managing blood pressure in stroke patients using a diverse sample: Integrating the person-based approach and patient and public involvement | Tanvi rai phd 2020 | UK | To describe the optimization of a digital intervention for stroke patients and the value of participant diversity, using the person-based approach (pba) and integral patient and public involvement (ppi) | Stroke patients and health-care professionals | N=40, 16women and 24 men ages ranged from 35 to over 80 stroke patients with varying degrees of post-stroke impairment, including aphasia, mobility issues, and diverse socio-economic and ethnic backgrounds. | Data were analyzed using the person-based approach (pba), with iterative cycles of data collection, analysis, and intervention modification. | Primary care and community settings in england and ireland | Digital self-monitoring and management of blood pressure (bp) | the Stroke Association and British Heart Foundation |
| 11.Older adult stroke survivors’ needs and perspectives of a stroke app: A qualitative study | Wenjing cao 2025 | China | To describe the needs and perspectives of older adult stroke survivors regarding a stroke app | Older adult stroke survivors | N=13, 5 women and 8 men  older adults (≥60 years), post-stroke, mobile phone users, no major cognitive or communication impairments. | Face-to-face semi-structured interviews; haase’s adaptation of colaizzi’s phenomenological method. | Three tertiary hospitals in chenzhou, hunan province, china; participants were community-dwelling older adults | Mobile health (mhealth) apps for stroke management | School-level scientific research project and Natural Science Foundation |
| 12.A qualitative study on user acceptance of a home-based stroke telerehabilitation system | Yu chen, 2019 | United states | To investigate patient perceived benefits of and barriers to using the telerehabilitation system at home | Patients in the subacute phase after stroke. | N=13, 2women and 11men age ≥18 years at the time of randomization 2.stroke that is radiologically verified, due to ischemia or to intracerebral hemorrhage | In-depth semi-structured interviews; thematic analysis based on the utaut model | Home-based rehabilitation | Home-based telerehabilitation system for stroke (including games, exercises, education, and videoconferencing) | National Science Foundation and National Institutes  of Health |
| 13.Exploring the need for mobile application in stroke management by informal caregivers: A qualitative study | Muhammad iqbal haji mukhti,2022 | Malaysia | To explore the need for a mobile application in stroke management by informal caregivers. | Informal caregivers of stroke survivors | N=13, 8women and 5men  the caregiver must be the stroke patient’s spouse, another family member, or a relative who is at least 18 years old and open to sharing their experiences. | In-depth interviews; thematic analysis (braun & clarke six-phase method) | Two main public rehabilitation centers involved | Mobile health (mhealth) applications for stroke management | Newton Ungku-Omar Fund of  the Ministry of Higher Education Malaysia |
| 14.Physiotherapists’ and occupational therapists’ perspectives on information and communication technology in stroke rehabilitation | Mille nabsen marwaa,2020 | Denmark | To explore the current and potential use of information and communication technology (ict) to enhance coherent person-centred rehabilitation after stroke, from the perspectives of physiotherapists and occupational therapists. | Physiotherapists and occupational therapists involved in stroke rehabilitation | N=9, 8women and 1men | Semi-structured interviews constructivist grounded theory approach with constant comparative method, initial coding, focused coding, and memo-writing. | The participants in this study were recruited from two regional hospitals (rehabilitation phases 1 and 2) and from two rural municipalities, i.e. A geographic area located outside town and cities with low population density (rehabilitation phase 3) in the region of south denmark. | Information and communication technology (ict) | University College South Denmark |
| 15. Experiences with mobile health-enabled ambulatory monitoring among stroke survivors: A qualitative study | Stephen c.l. Lau 2024 | USA | To identify facilitators and barriers for ambulatory monitoring among stroke survivors, as well as their suggestions for development and implementation of ambulatory monitoring. | Community-dwelling stroke survivors. | N=40, 17women and 23men  age 18 to 65 years,mild to moderate stroke (nih stroke scale ≤15),≥3 months poststroke,premorbid modified rankin scale ≤2 | Individual semi-structured interviews thematic content analysis using a preliminary codebook | Community-dwelling stroke survivors in the usa. | Mobile health (mhealth) technologies for ambulatory monitoring | Program in Occupational Therapy Dissertation Fund, Washington University |
| 16. Detecting user experience issues from mhealth apps that support stroke caregiver needs: An analysis of user reviews | Elton h. Lobo,2023 | USA | To utilize published user reviews of commercially available apps to determine the user experience issues to guide future app development in stroke caregiving. | Stroke caregivers | Users who posted reviews of mhealth apps for stroke caregivers no other information mentioned | Automated data capture，machine learning clustering+manual topic classification | Google play store, apple app store | Mhealth apps， specifically those designed to support stroke caregivers | Deakin University, and University of Copenhagen |
| 17. Acceptability of two mobile applications to support cross-sectoral, person-centred and empowering stroke rehabilitation – a process evaluation | Mille nabsen marwaa,2024 | Denmark | To evaluate the acceptability of two co-designed mobile applications mit sygehus [a knowledge-based solution] and genoptræn.dk [a self-training solution] to support a cross-sectoral, person-centred and empowering stroke rehabilitation | Stroke survivors | N=17, 8women and 9men  stroke survivors: 50-84 years old; important others: 50-78 years old | Individual and dyadic interviews  a constructivist grounded theory analysis | Two stroke rehabilitation trajectories in southern denmark | Mobile applications | University College of South Denmark and the Danish Organization |
| 18. Arm activity feedback for stroke survivors – a user needs analysis for the design of a mobile application | Laura mayrhuber 2025 | Switzerland | To improve user acceptance of both the app and the tracking device, thereby promoting consistent adherence to ul therapy at home. | Stroke survivors" And "Healthcare professionals (hcps) | N=8, 3women and 5men above 18 years of age,sub-acute or chronic stage ,residual unilateral hemiparesis | Semi-structured one-on-one interviews reflexive thematic analysis | At the cerebro - center for neurology & rehabilitation (viznau/ hertenstein, switzerland) | A smartwatch and -phone application (app) | Not report funding source |
| 19. Experiences of f@ce – a team-based, personcentred intervention supported by information and communication technology for rehabilitation after a stroke | Mandana fallahpour 2025 | Sweden | To explore the experiences of participation in f@ce, a team-based, person-centred intervention program supported by information and communication technology, among persons with stroke, their significant others, and the team members. | Persons with stroke, their significant others (partners, sons, daughters, or friends), and rehabilitation team members (occupational therapists and physiotherapists). | N=22, 16women and 6 men clients: Mean 64.8 years (range 44–78) significant others: Mean 53.3 years (range 43–71) team members: Not specified | Individual interviews qualitative content analysis | Conducted in stockholm, sweden, within inpatient and primary care rehabilitation settings. | Information and communication technology (ict) | Karolinska Institute |
| 20. Occupational therapists’ experiences of using a new internet-based intervention - a focus group study | Ida-maria barchéus 2024 | Sweden | To explore how occupational therapists experienced the use of a new internet-based intervention, ‘strategies for empowering activities in everyday life’ (see), focusing on supporting client resources to manage an active everyday life after stroke. | Occupational therapists (ots) | N=4, 3women and 1 men not specified in the provided text. | Four focus group sessions conducted over 22 months via video meetings. Qualitative content analysis | Conducted in outpatient rehabilitation clinics in northern sweden. | Internet-based intervention, specifically the “strategies for empowering activities in everyday life | the Kamprad  Family Foundation for Entrepreneurship |
| 21. Patient experience using digital therapy “vigo” For stroke patient recovery: A qualitative descriptive study | Klinta epalte 2023 | Latvia | To explore the patient experience of use of the digital assistant “vigo” For stroke recovery. | Patients with stroke | N=12, 7women and 5men  at least 18 years of age no previous record of ischemic or hemorrhagic stroke | Semi-structured telephone interviews deductive thematic analysis of interview transcripts | Stroke patients who received in-patient or out-patient rehabilitation after stroke. | Digital therapeutic application “vigo” – a computer-generated artificial intelligence-based application with conversational chatbot and gamification elements, designed for stroke recovery. | European Regional Development Fund project |
| 22. Perspectives of people with stroke, caregivers and healthcare professionals on an adaptive mhealth intervention for physical activity in the prevention of secondary stroke: A qualitative study | Nathan cardy 2024 | Ireland | To explore the perspectives of people with stroke, their caregivers and healthcare providers on the design and delivery of an adaptive, personalised mhealth intervention to promote physical activity after stroke. | People with stroke, caregivers, and healthcare professionals. | N=28, the gender ratio is not mentioned aged 18 or over, provide care (paid or unpaid), support or assistance to people post-stroke member of their professional body | Semi-structured one-to-one or focus group interviews reflexive thematic analysis following braun and clarke’s six-step approach | Conducted in ireland. Participants recruited from hospital and community/professional groups. | Mobile health (mhealth) interventions | Health Research Board, Ireland |
| 23. Co‐designing a digital stroke prevention platform: Leveraging lived experience and expert advice | Tara purvis 2025 | Australia | To co-design the ‘love your brain’ digital platform, including an online education program (massive open online course, mooc) and text messaging system, for community stroke prevention education and management. | Community members (with or without a history of stroke) and health knowledge experts (e.g., health professionals and researchers) | N=22, community members: 58% female,health knowledge experts: 80% female | Seven online focus groups per cohort (total 14), plus one final evaluative focus group per cohort after testing analysis: Inductive and deductive thematic analysis | Conducted online across australia. | Digital health platform comprising a massive open online course (mooc) and a text messaging system. | Australian Government Medical Research Future Fund |
| 24. A usability evaluation framework for a mobile application in supporting home-based rehabilitation for stroke patients: A qualitative study | Shih-ying chien 2025 | China | To determine the usability, satisfaction, and performance levels of a mobile app designed for the clinical monitoring of stroke patients | Stroke patients and healthcare professionals (rehabilitation physicians, physical therapists, occupational therapists) | N=20, 9women and 11men  median age of 48 years；average age of over 60 years；62 years (±5 years) | Mixed-methods: Usability tests, interviews, surveys. Audio recordings transcribed and analyzed with nvivo 12.0. | Conducted in a rehabilitation medical institution in taoyuan, taiwan; | Mobile health application | Not report funding source |

2.2.	Quantitative studies（8）

| **Title** | **Author，year** | **Country** | **Aim** | **Target population** | **Participants** | **Data collection and analysis** | **Setting** | **DHT(Type )** | **Funding** |
| --- | --- | --- | --- | --- | --- | --- | --- | --- | --- |
|  |  |  |  |  | **Sample size、female，Age、Characteristics** |  |  |  |  |
| 1. Health knowledge after stroke in Switzerland: a survey among health professionals on current practice and suggestions for the implementation of a technology-based educational program for stroke survivors | Giada, 2024 | Switzerland | To investigate the estimated level of knowledge of stroke patients regarding their disease, the methods of information commonly used in clinical practice, the topics that should be included in an educational program aimed at improving health knowledge among stroke survivors, and how such a program could be delivered with the help of technology-based education | Health Professionals | N=94, NG, NG, healthcare pro-fessionals working in Swiss clinics and hospitals in which stroke patients are admitted and treated | Online survey software QuestionPro. Descriptive statistics (mean (std)) and histograms. | Healthcare professionals were convenience sampled from Swiss clinics and hospitals | The survey included Mobile applications, unsupervised therapy systems, telehabilitation, technology-assisted education. | National  Research Foundation, Prime Minister’s Office, Singapore |
| 2. Knowledge and Perspectives of Community Members on Risk Assessment for Stroke Prevention using mobile health approaches in Nigeria | Sarfo, 2023 | Nigeria | To assess the knowledge of community dwelling adults on stroke risk and their willingness to use mobile health (mHealth) technology in assessing their stroke risk. | Community stroke-free persons | N=486, female=260(53.5%), age=47.4±15.5 years. Community stroke-free persons200 participants from Ibadan, 36 from Abeokuta and 250 from Zaria. Over 44% of participants had attained tertiary or higher level of education, and most were employed | Survey questionnaire,Descriptive statistics+inferential statistics | Nigeria Community Center | The study was about digital platforms such mHealth Apps and stroke risk calculator mobile phone application. | National  Research Foundation, Prime Minister’s Office, Singapore |
| 3. Acceptability and Attitude towards a Mobile-Based HomeExercise Program among Stroke Survivors and Caregivers: A Cross-Sectional Study | Mahmood, 2019 | India | To assess the acceptability and attitude towards a mobile-based home exercise program among stroke survivors and their primary caregivers. | Adult stroke survivors and caregivers | N=102 (included 50 patients and 52 caregivers), female=14(in patients)/32(in caregivers), age=55.2±13.39(in patients)/39.78±12.62(in caregivers). Adult stroke survivors who were medically stable, had functional communication abilities, and were able to comprehend either regional language (Kannada) or English. The caregivers who primarily took care of stroke survivors and were fluent in either regional language or English. Both stroke survivors and the caregivers use mobile phones. | Cross sectional questionnaire. descriptive statistics | Survivors and Caregivers were purposive sampled in a tertiary care university teaching hospital of the semiurban region of coastal southern India. | The study was about mobile-based home exercise program (mHealth) | Not report funding source |
| 4. Application of immersive virtual reality for assessing chronic neglect in individuals with stroke: the immersive virtual road-crossing task | Belger, 2024 | Germany | To identify and quantify discrete neglect symptoms in chronic stroke patients | chronic stroke patients and healthy participant | N=60 (20 for USN+, 20 for USN-, 20 for HC), female=20(5 in USN+, 8 in USN-, 7 in HC), age=60±6.93(USN+)/57.34±9.34(USN-)/59.05±9.52(HC). USN+: Chronic right-hemisphere post-stroke patients with chronic left-sided spatial neglect. USN-: Chronic right-hemisphere post-stroke patients without neglect. HC: healthy participant | Survey questionnaire, descriptive statistics+inferential statistics | Purposive Sampled from the Clinic for Cognitive Neurology, University Hospital Leipzig, Germany, and the Max Planck Institute for Human Cognitive and Brain Sciences, Leipzig, German | The study was about immersive Virtual Reality. | European Social Fund International Max Planck Research School |
| 5. Cognitive Training Using Virtual Reality: An Assessment of Usability and Adverse Effects | Bang, 2024 | Republic of Korea | To evaluate the usability and adverse effects associated with virtual reality (VR) cognitive training and identify factors influencing them | rehabilitation professional and patients with stroke | N=30 (20 for professionals and 10 for patients), female=12(professionals)/8(patients), age=30±4.8 (professional)/64.1±13.6 (patients). Patients with stroke were those aged ≥19 years, with a mini-mental state examination (MMSE) score ≥20, excluded history of seizures and severe dizziness, visual or hearing impairments, problem with neck movement, difficulty maintaining a seated position, and the anticipation of severe VR sickness. | VR training data and standardized questionnaires, descriptive statistics+inferential statistics | Rehabilitation Medicine Department of Yishan Hospital under the National Health Insurance Corporation of South Korea | The study was about immersive Virtual Reality. | National Rehabilitation  Center, Ministry of Health and Welfare, Korea |
| 6. Design and usability evaluation of an immersive virtual reality mirrored hand system for upper limb stroke rehabilitation | Hsieh, 2025 | Taiwan, Province of China | To develop an immersive virtual reality (VR) mirrored-hand system with real-life activities designed for the upper limb rehabilitation of people with stroke and to evaluate its clinical usability and feasibility. | individuals with stroke | N=15, female=3, age=53.20±6.73.  (1) diagnosis of unilateral stroke; (2) age between 20 and 80 years; (3) baseline Fugl-Meyer Assessment of the Upper Extremity (FMA-UE) score between 20 and 60; (4) ability to follow the study instructions and provide verbal feedback on user experiences (a score of ≥ 24 on the Mini-Mental State Examination, 2nd Edition: Standard Version [MMSE-2:SV]); and (5) normal or corrected-to-normal vision. | VR core data and questionnaire survey，descriptive statistics | Individuals were convenience sampled from Chang Gung Memorial Hospital and Taipei Hospital, Taiwan Province of China | The study was about immersive Virtual Reality and mirror therapy | National Science and Technology Council |
| 7. The Feasibility and User Experience of a Program of Progressive Cued Activity to Promote Functional Upper Limb Activity in the Inpatient Rehabilitation Setting with Follow-Up at Home | Bassindale, 2025 | USA | To promote upper limb activity in an inpatient rehabilitation facility (IRF) post-stroke | stroke survivors | N=30, female=12, NG. unilateral stroke within the last 30 days, age ≥ 18 years old, and English language speaking. Exclusion criteria included inability to give informed consent or follow two-stage instructions, severe cognitive impairment indicated by a score ≤9 on the Montreal Cognitive Assessment (MoCA), or concurrent illness or injury limiting the capacity to conform to study requirements. | Data collection, descriptive statistics+inferential statistics | Inpatient rehabilitation institutions and patient home environments in Wisconsin, USA | The study was about wearable activity monitor, mHealth and Remote wearable technology | The National Institutes of Health |
| 8. Unsupervised robot-assisted rehabilitation after stroke: feasibility, effect on therapy dose, and user experience | Devittori, 2024 | Switzerland | To explore the feasibility of unsupervised therapy with an upper limb rehabilitation robot in a clinical setting, evaluate the effect on the overall therapy dose, and assess user experience during unsupervised use of the robot and its usability | Stroke inpatients | N=13, female=3, age=65.9(49-77). inclusion within 6 weeks from stroke onset, pre-stroke modified Rankin score ≤ 1, National Institutes of Health Stroke Scale (NIHSS) ≥ 1 in at least one of the items regarding motor or sensory function and ataxia, and signed informed consent form. | Data collection, descriptive statistics, and inferential statistics | Stroke inpatients were convenience sampled from Clinica Hildebrand Centro di riabilitazione Brissago, Switzerland. | The study was about active end-effector device for hand rehabilitation, sensor-based device, exergame, unsupervised therapy, active (i.e., actuated) robotic device, irtual environment, robot and active rehabilitation robot. | Not report funding source |

2.3.	Mixed-methods studies（10）

| **Title** | **Author，year** | **Country** | **Aim** | **Target population** | **Participants** | **Data collection and analysis** | **Setting** | **DHT**  **(Type )** | **Funding** |
| --- | --- | --- | --- | --- | --- | --- | --- | --- | --- |
|  |  |  |  |  | **Sample size、female，Age、Characteristics** |  |  |  |  |
| 1. Multicomponent Support Program for Secondary Prevention of Stroke Using Digital Health Technology: Co-Design Study With People Living With Stroke or Transient Ischemic Attack | Silvera-Tawil, 2024 | Australia | To co-design, with people with lived experience of stroke or TIA (referred to as consumers) and clinicians, a multicomponent digital technology support program for secondary prevention of stroke | people living with stroke or TIA | N=112, female=45, age=71.94(40~98). diagnosis of stroke or TIA in the past 6 months to 5 years; aged ≥18 years; proficiency in spoken and written English; and, for AuSCR participants, those who agreed to be contacted by the AuSCR for future research | Descriptive statistics+inferential statistics+audio transcription and inductive coding | Communities and clinical institutions in Australia | the study includes mHealth, wearable devices, Clinician Web Portal and Electronic Message System | The Commonwealth  Scientific and Industrial Research Organisation and Monash University |
| 2. Adopting information and communications technology in the control, prevention, and management of stroke: perspectives from patients and providers in Uganda | Namyalo, 2024 | Uganda | to facilitate the design of a mobile phone app as an intervention | stakeholders including health workers and potential stroke patients/caregivers/survivor | N=19, NG | Extract stroke case data from 2015 to 2021 and conduct semi-structured interviews; Descriptive statistics, inductive method | All participants were recruited from , Kiwoko and Luwero Hospital, Uganda. | The study includes mHealth technologies such as mobile phone and applications. | Research and Innovation Department of Ndejje University |
| 3. Analysis, Design, and Prototypical Implementation of a Serious Game Reha@Stroke to Support Rehabilitation of Stroke Patients With the Help of a Mobile Phone | Baranyi, 2020 | Austria | To propose a new and innovative serious game, which relies only on integrated sensors of a smart-phone. | Therapists, stroke patients and medical doctors | N=19 (4 therapists, 13 patients, 2 medical doctors), NG, NG, NG | Interview, with a focus on qualitative analysis | The study was held by Research Group for Industrial Software, TU Wien, 1040 Vienna, Austria | The study includes mobile phone, Serious Game, mobile application, virtual reality application, sensors, User Centered Design. | Not report funding source |
| 4. Development of a Digital Lifestyle Modification Intervention for Use after Transient Ischaemic Attack or Minor Stroke: A Person-Based Approach | Heron, 2021 | UK | To explore perceptions on usability and relevance of the app in order to maximise user engagement and sustainability | CVD patients | N=32, female=21, NG. All participants had experienced a Transient Ischaemic Attack or stroke at least one month but no more than 2 years prior to recruitment | Database search+focus group; Inductive thematic analysis method+construction of intervention model | Participants were purposive sampled from Northern Ireland Chest Heart and Stroke, UK | The study includes mHealth technologies such as health management module, behavior change technology integration, mobile device platform and remote support integration. | National Institute of Health Research |
| 5. Development of a Digital Tool for People With a Long-Term Condition Using Stroke as a Case Example: Participatory Design Approach | Kjörk, 2022 | Sweden | to develop a digital previsit tool and explore potential end user’s perceptions, using a participatory approach with stroke as a case example | Staffs from an outpatient unit, Stroke Forum, and a support association | N=22, female=9, age=59(42~83). Staff identified eligible individuals in 3 settings: an outpatient unit, Stroke Forum (a center for support and advice after initial care), and a support association. Potential participants were briefly informed about the study, and those who agreed to participate were contacted | Focus group, questionnaire survey; Framework method, descriptive statistics | All the participants were purposive sampled from an outpatient unit, Stroke Forum, and a support association, Sweden | The study includes mHealth technologies such as Digital preview tool and User portraits. | In part by grants from the Swedish Science Council |
| 6. mHealth intervention for carers of individuals with a history of stroke: Heuristic evaluation and user perspectives | Lobo, 2022 | Australia | To perform a heuristic evaluation of mHealth interventions designed to support carers of individuals with a history of stroke and determine the user preferences in stroke caregiving technology to inform future researchers and developers regarding the best practices to support these individuals. | Usability experts and Carers | N=20, 10(Experts)/10(Carers), female=3(Experts)/7(Carers), age=34.6(30~49, Experts) /41.7(24~61, Carers). More than 1 year of availability assessment experience and have completed more than 20 similar tasks. (Experts) Cared for individuals with a history of stroke in the past 5 years. (Carers) | Questionnaire investigation; Grounded theory, descriptive statistics | Experts were purposive sampled from Amazon Mechanical Turk, Australia. Carers were purposive sampled from Carers VIC and Carers QLD, Australia. | The study includes mHealth Technologies such as Personalized information recommendation system, Multi-dimensional monitoring, Health Tracking Module and Mixed mobile intervention model | No financial support |
| 7. Novel Multicomponent Digital Care Assistant and Support Program for People After Stroke or Transient Ischaemic Attack: A Pilot Feasibility Study | Allan, 2024 | Australia | To determine the feasibility of the novel Care Assistant and support Program for people after Stroke (CAPS) or transient ischaemic attack (TIA) by combining person-centred goal setting and risk-factor monitoring through a web-based clinician portal, SMS messages, a mobile application (app), and a wearable device. | people living with stroke or TIA and clinicians involved in the potential future delivery of the program | N=33, female=9, age=70.5(53.9,78.7). Had a diagnosis of stroke or TIA in the previous 6 months to 3 years, were aged ≥ 18 years old, were living in the community, were proficient in spoken and written English, and had access to a smartphone with internet access | Equipment data collection, semi-structured interviews; Descriptive statistics, inductive thematic analysis | Australian Community | The study includes mHealth technologies such as cloud-based mobile technology-enabled rehabilitation platform, multicomponent digital health program and synchronised wearable device. | Commonwealth Scientific and Industrial Research Organisation Precision Health Future Science Platform |
| 8. Usability and feasibility of PreventS-MD web app for stroke prevention | Feigin, 2024 | New Zealand | Evaluate usability and feasibility of PreventS-MD by HCPs and individuals at risk of stroke/CVD. | Health Care Professionals | N=68, female=25(Phase1)24(Phase2), age=NG. HCPs who were English-speaking and of any age, sex, race/ethnicity, and geographical locality were invited via circulating emails through the networks of the World Stroke Organization (WSO) and the National Institute for Stroke and Applied Neurosciences at Auckland University of Technology. | Semi structured interviews and questionnaire surveys; Descriptive statistics, thematic analysis | All participants were purposive sampled from 27 countries | The study includes mHealth technologies such as Digital Support System, Stroke Riskometer and web app. | No financial support |
| 9. User-Centered Mobile Applications for Stroke Survivors (MAPPS): A Mixed-Methods Study of Patient Preferences | Ramaswamy, 2023 | USA | to provide a user-centered framework for an mHealth app for SS by identifying their preferences for the app’s features and studying the perceived usefulness of these features. | Stroke survivors | N=1194, female=686, age=62.3±12.9. | Focus groups, semi-structured interviews, questionnaire surveys; Descriptive statistics, thematic analysis | All participants were convenience sampled from our hospital stroke centers, adult day programs, stroke advocacy networks, support groups, and the National Stroke Association (NSA) database in USA. | The study includes mHealth technologies such as mHealth app, User Centered Design, Data Tracking and Management, Accessible Design and Interactive technology. | The Patient-Centered Outcomes Research Institute |
| 10. Post-intervention qualitative assessment of mobile health technology to manage hypertension among Ghanaian stroke survivors | Nichols, 2019 | Ghana | To explore post-intervention perspectives and gather in-depth insight into the experiences of stroke survivors and their caregivers following participation in a larger interventional arm of a mHealth blood pressure management study | Stroke survivors and caregivers. | N=31, 16(stroke survivors)/8(caregivers)/7(research team), female=8(participants)/3(nonparticipants), age=54.3±11.6(participants)/54.4±12.8 (nonparticipants).  Survivors: recent stroke patients, uncontrolled hypertension | Focus group, equipment data collection; thematic content analysis | All participants were convenience sampled from Komfo Anokye Hospital, Kumasi, Ghana | The study includes mHealth technologies such as Phone-based Intervention, Automatic collection of blood pressure data, Data visualization feedback, Remote data monitoring and Clinical support platform. | The National  Institutes of Health-National Institute |
