# Supplementary material for: Stakeholder Perspectives on Digital Health Technologies in Poststroke Self-Management and Rehabilitation: Systematic Review Based on the NASSS Framework
Source: JMIR Mhealth Uhealth. 2026 Oct 9;14:e95197. doi: 10.2196/95197 (PMC13656741; doi:10.2196/95197)
Supplement: Supplementary file 3 [file mhealth-v14-e95197-s003.docx]

Stakeholder Perspectives on Digital Health Technologies in Post-Stroke Self-Management and Rehabilitation: A Systematic Review Based on the NASSS Framework

**Appendix 3.**

Quality appraisal.

Quality assessment of included studies using the Mixed-Methods Appraisal Tool, stratified by study design

3.1.	Qualitative studies

|  | **Screening questions** | | **Qualitative** | | | | |
| --- | --- | --- | --- | --- | --- | --- | --- |
| First author**，**Year | S1. | S2. | 1.1 | 1.2 | 1.3 | 1.4 | 1.5 |
| Esmee L S Bally，2023 | Y | Y | Y | Y | Y | Y | Y |
| Esther M. G.，2022 | Y | Y | Y | Y | Y | Y | Y |
| Brigid Clancy,2024 | Y | Y | Y | Y | Y | Y | Y |
| Ann Marie Hestetun-Mandrup,2025 | Y | N | N | N | N | N | N |
| Henna Härkönen,2024 | Y | Y | Y | Y | N | Y | Y |
| Kathryn Jarvis,2025 | Y | Y | Y | Y | Y | Y | Y |
| Dinah Amoah,2023 | Y | Y | Y | Y | Y | Y | Y |
| Gerardo Luis Dimaguila,2020 | Y | Y | Y | N | Y | Y | Y |
| Tom Smejka,2022 | Y | Y | N | Y | Y | Y | Y |
| Tanvi Rai PhD，2020 | Y | Y | Y | Y | Y | Y | Y |
| Wenjing Cao,2025 | Y | Y | Y | Y | Y | Y | Y |
| Yu Chen，2020 | Y | Y | Y | Y | Y | Y | Y |
| Muhammad Iqbal Haji Mukhti,2022 | Y | Y | Y | Y | Y | Y | Y |
| MilleNabsenMarwaa，2020 | Y | Y | Y | Y | Y | Y | Y |
| Stephen C. L. Lau,2025 | Y | Y | Y | Y | Y | Y | Y |
| Elton H. Lobo, 2023 | Y | Y | Y | Y | Y | Y | Y |
| Mille Nabsen Marwaa,2024 | Y | Y | Y | Y | Y | Y | Y |
| Laura Mayrhuber,2025 | Y | Y | Y | Y | Y | Y | Y |
| Mandana Fallahpour,2025 | Y | Y | Y | Y | Y | Y | Y |
| Ida-Maria Barchéus,2024 | Y | Y | Y | N | N | Y | Y |
| Klinta Epalte,2020 | Y | Y | Y | Y | Y | Y | Y |
| Nathan Cardy,2024 | Y | Y | Y | Y | Y | Y | Y |
| Tara Purvis，2025 | Y | Y | Y | Y | Y | Y | Y |
| Shih-Ying Chien，2025 | Y | Y | Y | Y | Y | Y | Y |

3.2.	Quantitative studies

|  | **Screening questions** | | | **Quantitative descriptive** | | | |
| --- | --- | --- | --- | --- | --- | --- | --- |
| **First author，Year** | S1. | S2. | 4.1. | 4.2. | 4.3. | 4.4 | 4.5 |
| Giada Devittori,2024 | Y | Y | Y | N | Y | Y | Y |
| Fred Stephen Sarfo,2023 | Y | Y | C | Y | Y | Y | Y |
| Amreen Mahmood,2019 | Y | Y | Y | N | Y | Y | N |
| Julia Belger,2024 | Y | Y | Y | Y | Y | Y | Y |
| Myeonghwan Bang, MD,2024 | Y | Y | Y | N | Y | C | Y |
| Yu-Wei Hsieh,2025 | Y | Y | N | Y | Y | C | Y |
| Kimberly Bassindale,2025 | Y | Y | C | Y | Y | N | Y |
| Giada Devittori,2024 | Y | Y | Y | N | Y | Y | Y |

3.3.	Mixed-methods studies

|  | **Screening questions** | | **Qualitative** | | | | | **Quantitative descriptive** | | | | | **Mixed methods** | | | | |
| --- | --- | --- | --- | --- | --- | --- | --- | --- | --- | --- | --- | --- | --- | --- | --- | --- | --- |
| **First author，Year** | S1. | S2. | 1.1 | 1.2 | 1.3 | 1.4 | 1.5 | 4.1 | 4.2 | 4.3 | 4.4 | 4.5 | 5.1 | 5.2 | 5.3 | 5.4 | 5.5 |
| David Silvera-Tawil1,2024 | Y | Y | Y | Y | Y | Y | Y | Y | Y | Y | Y | Y | Y | Y | Y | Y | Y |
| Prossy Kiddu Namyalo,2024 | Y | Y | Y | Y | N | Y | Y | Y | Y | Y | Y | Y | Y | Y | N | Y | Y |
| René Baranyi,2020 | Y | Y | Y | Y | Y | Y | Y | Y | N | Y | Y | Y | Y | Y | Y | Y | Y |
| Neil Heron，2021 | Y | Y | Y | Y | Y | Y | Y | Y | Y | Y | Y | Y | Y | Y | N | Y | Y |
| Emma K Kjörk，2022 | Y | Y | Y | Y | Y | Y | Y | Y | Y | Y | Y | Y | Y | Y | Y | Y | Y |
| Elton H Lobo，2022 | Y | Y | Y | Y | Y | Y | Y | Y | Y | Y | Y | Y | Y | Y | N | Y | Y |
| Liam P. Allan，2024 | Y | Y | Y | Y | Y | Y | Y | Y | N | Y | N | Y | Y | Y | N | Y | Y |
| Valery L Feigin，2024 | Y | Y | Y | Y | Y | Y | Y | Y | Y | Y | Y | Y | Y | Y | Y | Y | Y |
| Srinath Ramaswamy,2023 | Y | Y | Y | Y | Y | Y | Y | Y | Y | Y | N | Y | Y | Y | N | Y | Y |
| Michelle NicholS,2019 | Y | Y | Y | Y | Y | Y | Y | N | N | N | N | N | Y | Y | N | N | N |

S1. Are there clear research questions? S2. Do the collected data allow to address the research questions?

1.1. Is the qualitative approach appropriate to answer the research question? | 1.2. Are the qualitative data collection methods adequate to address the research question? | 1.3. Are the findings adequately derived from the data? | 1.4. Is the interpretation of results sufficiently substantiated by data? | 1.5. Is there coherence between qualitative data sources, collection, analysis and interpretation?

4.1. Is the sampling strategy relevant to address the research question? | 4.2. Is the sample representative of the target population? | 4.3. Are the measurements appropriate? | 4.4. Is the risk of nonresponse bias low? | 4.5. Is the statistical analysis appropriate to answer the research question?

5.1. Is there an adequate rationale for using a mixed methods design to address the research question? | 5.2. Are the different components of the study effectively integrated to answer the research question? | 5.3. Are the outputs of the integration of qualitative and quantitative components adequately interpreted? | 5.4. Are divergences and inconsistencies between quantitative and qualitative results adequately addressed? | 5.5. Do the different components of the study adhere to the quality criteria of each tradition of the methods involved?

Y = Yes, N = No, C = Can’t tell
